# What secondary research evidence exists on the effects of forest management after disturbances: a systematic map protocol

**DOI:** 10.1186/s13750-024-00340-7

**Published:** 2024-06-02

**Authors:** Moritz Baumeister, Markus A. Meyer

**Affiliations:** 1 Department of Agriculture, Ecotrophology, and Landscape Development, National and International Nature Conservation, University of Applied Sciences Anhalt, Bernburg, Germany; 2 Ecosystem Modelling, Faculty of Forest Sciences and Forest Ecology, University of Göttingen, Göttingen, Germany

**Keywords:** Salvage logging, Afforestation, Tree planting, Biodiversity, Ecosystem services, Natural regeneration, Restoration, Evidence synthesis, Evidence review map, Meta-analysis

## Abstract

**Background:**

Forest disturbances are projected to increase in intensity and frequency in the upcoming decades. The projected change in disturbance regimes is expected to alter the provision of ecosystem services and affect biodiversity. Both are critical for forest ecosystems to provide livelihoods for human societies. Forest management after natural disturbances shapes successional pathways of forest ecosystems. Therefore, the management of post-disturbance sites deserves critical attention to avoid negative effects of management interventions on ecosystem services and biodiversity. The two most common management interventions after natural disturbances are salvage logging (comparator: no salvage logging) and tree planting (comparator: natural regeneration). This planned systematic map of reviews aims to aggregate the existing evidence syntheses on the implications of common forest management interventions after natural disturbances on successional trajectories with regard to selected ecosystem services and biodiversity. Evidence-based post-disturbance management is highly relevant for protected area management as well as for the management of commercial forests.

**Methods:**

We will systematically search the databases Scopus, Web of Science Core Collection and the Forest Science Collection of the CABI Digital Library for reviews and meta-analyses (after 2003). We will apply eligibility criteria for review selection and assess the evidence synthesis validity of selected reviews using the most recent version of CEESAT (Collaboration for Environmental Evidence Synthesis Assessment Tool). The results will be displayed in topic subgroups in summary of scope and summary of findings tables.

**Supplementary Information:**

The online version contains supplementary material available at 10.1186/s13750-024-00340-7.

## Background

Natural disturbances structure ecosystems worldwide. Due to climate change, rapid shifts in abiotic conditions are expected to change global disturbance regimes and thus alter ecosystems [1]. In forest ecosystems, disturbances are projected to be more frequent and intense in the future and also the likelihood of interactions of different disturbance types is expected to increase [2].

An increase in disturbance frequencies and intensities in forests also changes the provision of ecosystem services [3] and affects biodiversity [4]. Species diversity and, in particular, the functional diversity of species are closely linked to the ability of ecosystems to provide a portfolio of ecosystem services [5]. Thus, the rapidly changing world causing biodiversity loss [6, 7] and increased disturbance intensities and frequencies result in great uncertainties regarding the provision of ecosystem services [8]. Human societies, however, are inseparably intertwined with their surrounding ecosystems [9] and are heavily dependent on a reliable provision of ecosystem services [10].

Forest management has the potential to mitigate negative disturbance impacts on ecosystem service provision [2]. After natural disturbances, current forest management is usually guided by two silvicultural management questions: Should forest management opt (i) for salvage logging [11] and (ii) for planting trees [12]? These two simple management questions entail more complex follow-up questions. For instance, the potential expansion of green tree retention harvest procedures [13, 14] to harvest procedures after natural disturbances [15].

In forestry, however, the evaluation of management decisions can only be evaluated after long time periods *ex post* [16]. Forest management has long-lasting and often unforeseen legacies far into the future [17] and may fundamentally affect the successional trajectories of forests [18]. Therefore, adequate forest management requires permanent and dynamic adaptation and evaluation of management decisions [19] and needs to embrace the complexity of forest ecosystems instead of seeing it as an obstacle to achieving clear management targets [20].

To embrace the complexity of forest ecosystems and to acknowledge the fact that we can never predict ecosystem dynamics over decades without considerable uncertainties strengthens the importance of forest management grounded in available ecological knowledge. Furthermore, it highlights the potential for severe consequences of incautious management decisions. To link management decisions with their consequences for ecosystems, systematic reviews and meta-analyses have been established as tools in ecology to aggregate the findings of individual studies as part of the “evidence-based conservation movement” [21, 22]. Systematic reviews and meta-analyses provide a powerful tool to guide forest management and estimate the impacts of certain management interventions on forest ecosystems. For example, meta-analyses were conducted to assess the effects of salvage logging on ecosystem services [23], biodiversity in general [24] and specific taxa [25].

It has first been recognised in medical sciences that decision-makers and scientists face difficulty reading the large number of systematic reviews published regularly [26]. Therefore, a new article type called overview of reviews (sometimes also referred to as umbrella review, review of reviews, synthesis of systematic reviews or summary of systematic reviews [27]) was established in recent years, and its methods are still under development [28]. Despite the infancy of the overview methodology in medical sciences [29], a similar methodology to aggregate existing secondary research has also been developed under the term “evidence review mapping” in environmental sciences [30]. Here, we refer to our methodology as a systematic map of reviews to be consistent with the established terms by the Collaboration for Environmental Evidence (CEE).

Management decisions are usually made shortly after natural disturbances occur [11], and environmental managers are unlikely to have the time to aggregate the fragmented knowledge from many different reviews. Thus, adopting methods that allow to map the evidence from secondary research regarding the effect of management decisions after natural disturbances with respect to ecosystem services and biodiversity provides an accessible knowledge base for environmental managers and political decision-makers.

## Stakeholder engagement

The increased frequency and intensity of extreme weather events as well as an increase in the extent, frequency and impacts of pests and diseases in forest ecosystems have been identified as essential challenges for the supply of ecosystem services in Europe [31]. Understanding the influence of forest management on the successional trajectories of forest ecosystems is key to making careful and considerate forest management decisions that include a broad portfolio of ecosystem services and consider biodiversity conservation. The idea to provide a reader-friendly systematic map of reviews relevant to forest management after natural disturbances originated from the Wald-Klima-Forum (Forest-Climate-Forum) in Jena, Germany, in June 2022 and the conference “Holznutzung in Krisenzeiten” (Timber use in Times of Crisis) in Göttingen, Germany, in April 2023. On both occasions, stakeholders from various backgrounds (forestry sector, nature conservation organizations, scientists) discussed urgent questions of current forest management. One of the overarching questions was how to manage forest sites after natural disturbances. The debate is not new [32] but was reinforced due to recent widespread bark beetle outbreaks and prolonged summer droughts negatively affecting the main economic tree species in Central Europe [33]. The discussion intensified in the last few years, spread beyond the scientific community and became also highly relevant for the management of disturbance sites within protected areas such as national parks [34].

## Objective of the review

**Fig. 1:**
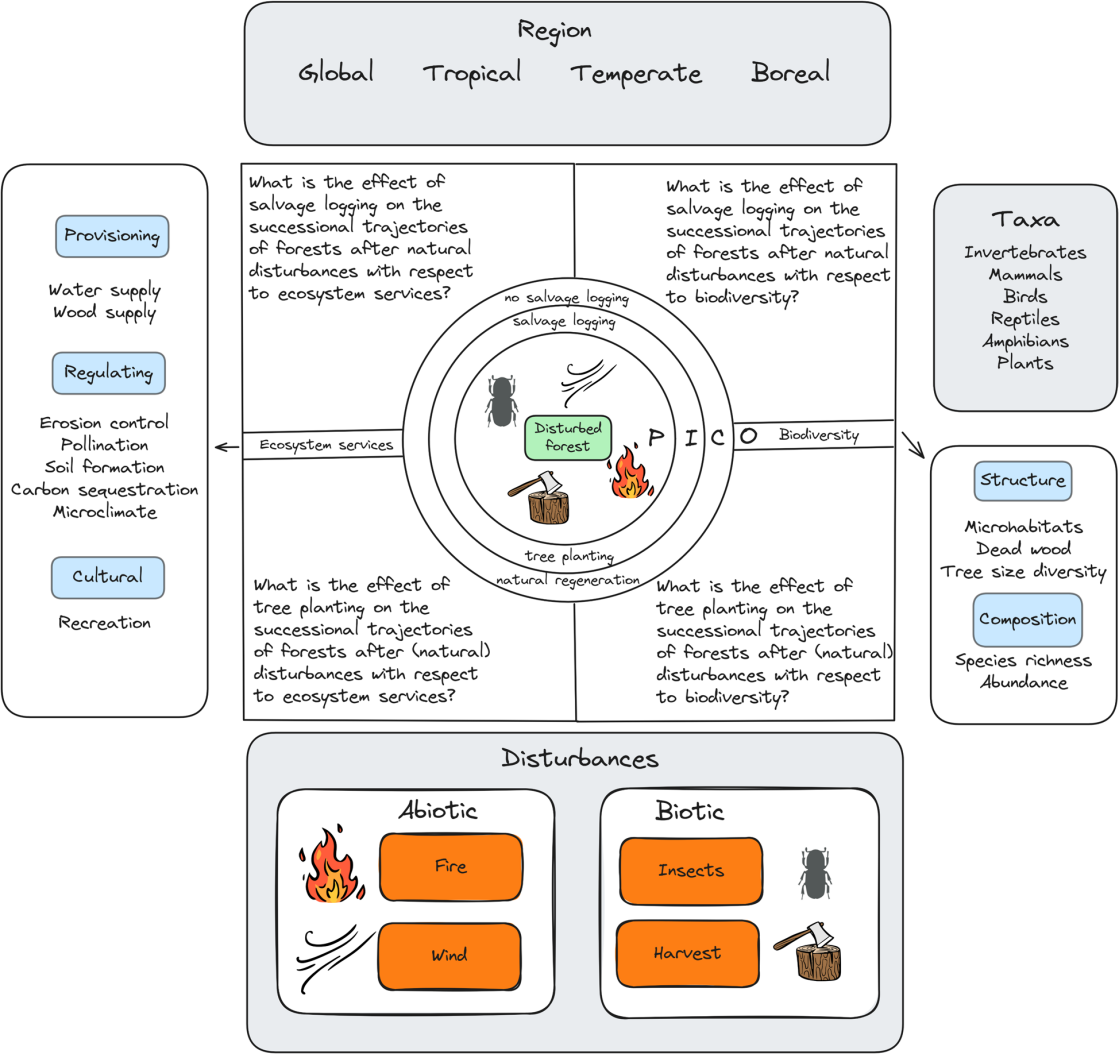
Detailed visualization of Population, Interventions, Comparators and selected Outcomes of interest for the planned systematic map. The structure follows the PICO elements for question formulation in evidence syntheses following the CEE guidelines [45]. Grey boxes indicate additional categories by which the mapped reviews will be grouped. References for the illustrations: cocomaterial (https://creativecommons.org/publicdomain/zero/1.0/); bark beetle © Dorota Paczesniak (https://creativecommons.org/licenses/by-sa/4.0/); Link: ; no changes made

The objective of our systematic map of reviews is to summarize the evidence of recent reviews (after 2003) addressing how common management interventions after forest harvesting and natural disturbances (salvage logging and tree planting) affect forest successional trajectories. This systematic map aims to aggregate the key findings of previous reviews to guide forest management with respect to selected ecosystem services and biodiversity (Fig. 1). Systematic maps of reviews act as an entry point for a more detailed examination of certain aspects of a topic [26] and should be written as user-friendly documents to reach a broad audience of scientists and environmental decision-makers [29].

Our selection of ecosystem services is based on the list of forest ecosystem services mapped by Orsi et al. 2020 for the European Union: Wood supply, water supply, erosion control, pollination, soil formation, climate regulation (carbon sequestration) and recreation [35]. We do not consider “habitat provision” from their list as we treat biodiversity as a separate entity within our analysis. Additionally, we consider forest microclimate regulation as an ecosystem service class [36]. We include microclimate because the effect of forest management on microclimate regulation and its potential to impact the climate on the landscape scale is a heavily debated current topic in forestry (e.g. [37]).

With respect to biodiversity, we explicitly consider categories related to forest structure that are indicators for biodiversity: tree size diversity, microhabitats and dead wood [38, 39]. We will further group our results into different taxa: Plants, invertebrates, mammals, birds, reptiles and amphibians [40].

For this systematic map of reviews, we additionally differentiate between different natural disturbance agents (fire, wind and insects) and the geographical scale of the respective review (Fig. 1). Even though the systematic map  is designed to summarize secondary evidence for forest management after natural disturbances, we decided to also include anthropogenic disturbances. Studies investigating successional trajectories after harvest may provide valuable insight for the questions at hand. Despite the focus on natural disturbance sites, excluding normal harvest procedures would decrease the body of evidence unjustifiably. Wood harvest is a very common anthropogenic disturbance and research on its effects on forest ecosystems has been conducted for decades [41].

The primary question is ‘What is the effect of salvage logging (comparator: no salvage logging) and/or tree planting (comparator: natural regeneration) on the successional trajectories of forests after natural disturbances and forest harvesting with respect to ecosystem services and biodiversity? For detailed question elements see Fig. 1.

## Methods

The general principles that guide the planning of a Systematic Map of primary studies are also applicable for mapping reviews[27]. Thus, this protocol is planned in accordance with the “ CEE Guidelines and Standards for Evidence Synthesis in Environmental Management” [42]. A completed ROSES form (reporting standards for systematic evidence syntheses) for systematic map protocols for this protocol is provided as supplementary material (Additional file 1 following [22]).

### Searching for articles

The search strings were defined based on population, intervention, and outcome [21, 43] and by comparison to existing systematic review protocols in the field of forest ecology [44–47]. The databases Scopus, Web of Science (core collection), and the Forest Science Collection of the CABI Digital Library will be searched (Table 1) for reviews and meta-analyses after 2003 in four distinct categories: (1) salvage logging and ecosystem services, (2) salvage logging and biodiversity, (3) tree planting (comparator: natural regeneration) and ecosystem services, (4) tree planting (comparator: natural regeneration) and biodiversity. These four categories result in four individual search strings (Table 2). As part of the methodology of systematically mapping reviews, the search intentionally aims to exclude primary research [28]. The search covers the two most widely used databases of the natural sciences [48] and a database with a forestry-specific collection. Additionally, the reference list of every fully read review will be screened for potentially relevant reviews that were not detected by the database search and we will also screen the websites of the following organizations for links or references to relevant publications and data, including grey literature:

**Table 1:** The proposed databases to search studies

Database	Institutional subscription	Search fields
Web of science core collection • WoS—Science Citation Index Expanded (SCIE) • Journal Citation Reports (InCites JHCD) • Social Sciences Citation Index (SSCI) •Medline	Göttingen University	Topic (includes title, abstract, author keywords, and key-words plus)
Scopus	Anhalt University	Article Title, Abstract, Keywords
CABI digital library • Forest science collection	Göttingen University	Article Title, Abstract, Keywords

**Table 2:** Search strings for the systematic map of reviews

Category	Search string
Salvage logging and ecosystem services	TITLE-ABS-KEY( (forest* OR woodl*) AND (disturb* OR succession* OR harvest* OR fire OR wildfire OR windthrow OR storm OR ((pest OR insect* OR beetle*) AND (outbreak OR attack))) AND (“snag remov*” OR ((salvag* OR post*) AND (log* OR harvest* OR cut* OR fell*))) AND (“ecosystem service*” OR “environmental service*” OR “ecosystem function*” OR “wood supply” OR “tree regenerat*” OR recov* OR “water supply” OR erosion OR pollinat* OR “soil formation” OR “climate regulation” OR “carbon sequestration” OR “carbon storage” OR recreat* OR microclimat*) AND (review OR “meta$analy*” OR synthes* OR datasets) ) AND PUBYEAR > 2003 AND LANGUAGE(english) AND SRCTYPE(j OR b)
Salvage logging and biodiversity	TITLE-ABS-KEY( (forest* OR woodl*) AND (disturb* OR succession* OR harvest* OR fire OR wildfire OR windthrow OR storm OR ((pest OR insect* OR beetle*) AND (outbreak OR attack))) AND (“snag remov*” OR ((salvag* OR post*) AND (log* OR harvest* OR cut* OR fell*))) AND (biodivers* OR diversity OR richness OR “species richness” OR “tree size diversity” OR microhabitat* OR “dead wood” OR invertebrat* OR vertebrat* OR flora OR vegetat* OR mammal* OR bird* OR avian OR reptile* OR amphibian*) AND (review OR “meta$analy*” OR synthes* OR datasets) ) AND PUBYEAR > 2003 AND LANGUAGE(english) AND SRCTYPE(j OR b)
Tree planting (comparator: natural regeneration) and ecosystem services	TITLE-ABS-KEY( (forest* OR woodl*) AND (disturb* OR succession* OR degrad* OR restorat* OR log* OR harvest* OR cut* OR fell* OR fire OR wildfire OR windthrow OR storm OR ((pest OR insect* OR beetle*) AND (outbreak OR attack))) AND (plant* OR seed* OR sowing OR “seed* establish*” OR grow* OR afforestat* OR restorat* OR “natural rejuvenation” OR regenerat* OR rejuvenat* OR “seed* establish*” OR seed* OR “young adj4 tree*”) AND (“ecosystem service*” OR “environmental service*” OR “ecosystem function*” OR “wood supply” OR “tree regenerat*” OR recov* OR “water supply” OR erosion OR pollinat* OR “soil formation” OR “climate regulation” OR “carbon sequestration” OR “carbon storage” OR recreat* OR microclimat*) AND (review OR “meta$analy*” OR synthes*)) AND PUBYEAR > 2003 AND LANGUAGE(english) AND SRCTYPE(j OR b)
Tree planting (comparator: natural regeneration) and biodiversity	TITLE-ABS-KEY( (forest* OR woodl*) AND (disturb* OR succession* OR degrad* OR restorat* OR replace* OR log* OR harvest* OR cut* OR fell* OR fire OR wildfire OR windthrow OR storm OR ((pest OR insect* OR beetle*) AND (outbreak OR attack))) AND (plant* OR seed* OR sowing OR “seed* establish*” OR grow* OR afforestat* OR restorat* OR “natural rejuvenation” OR regenerat* OR rejuvenat* OR “seed* establish*” OR seed* OR “young adj4 tree*”) AND (biodivers* OR diversity OR richness OR “species richness” OR “tree size diversity” OR microhabitat* OR “dead wood” OR invertebrat* OR vertebrat* OR flora OR vegetat* OR mammal* OR bird* OR avian OR reptile* OR amphibian*) AND (review OR “meta$analy*” OR synthes*) ) AND PUBYEAR > 2003 AND LANGUAGE(english) AND SRCTYPE(j OR b)

The search strings displayed are for the search in the database Scopus (the search strings for the other databases are provided as Additional file seven). If any later changes are made in the search strings, the adjustments are disclosed in the methods section of the final publication

European Environment Agency (http://www.eea.europa.eu).

Food and Agriculture Organization of the United Nations (http://www.fao.org).

International Union for Conservation of Nature (http://www.iucn.org).

Society for Ecological Restoration (http://www.ser.org).

International Union of Forest Research Organizations (https://www.iufro.org/

We do not search library databases to include grey literature. We acknowledge this as a potential limitation with respect to the comprehensiveness of our search.

The search terms will be searched within the title, abstract and keywords (TITLE-ABS-KEY). The search is restricted to research published in English as a journal article or as a book chapter (SRCTYPE) after 2003 (PUBYEAR > 2003, the year when the Centre for Evidence-based Conservation was established.). The final publication of the systematic map will include dates of the searches and the full search strings modified for the different databases.

Test searches for the intervention categories of tree planting and natural regeneration pointed out that the removal of the terms “(disturb* OR succession)” resulted in the inclusion of studies that investigated the respective interventions after usual harvest procedures (e.g., clear-cut forestry, retention forestry). Despite the focus on natural disturbances, we want our systematic map to include this well-studied disturbance agent. Mapping the reviews conducted for anthropogenic disturbances most likely provides valuable additional insights (see also section *Objective of the review* for reasoning).

### Review screening and review eligibility criteria

The search results will be imported into Excel and the removal of duplicates will be conducted using the statistical programming language R [49] within the integrated development environment RStudio. All search results within the four search categories will be screened based on predefined eligibility criteria (Table 3 after[50]) by two independent reviewers [51]. The inclusion or exclusion of studies follows the usual hierarchical approach of screening first only the title, second the abstract and third the full text of the respective review [50]. All hierarchical levels of the screening process will be managed with the software Excel.

**Table 3:** Eligibility criteria

Key element	Eligibility criteria	ID
Population Disturbed forest area	Included: explicit reference to a disturbed forest area (of boreal, temperate, tropical forest)	P
Intervention salvage logging, tree planting	Included: explicit reference to the interventions of interest	I
Comparator No salvage logging, natural regeneration	Included: explicit reference to the comparators of interest	C
Outcomes Ecosystem services *Water supply,* *Wood supply,* *Erosion control,* *Pollination,* *Soil formation,* *Carbon sequestration,* *Microclimate* *Recreation* Biodiversity *Invertebrates* *Mammals* *Birds* *Reptiles* *Amphibians* *Tree size diversity* *Microhabitats* *Dead wood*	Included: explicit reference to the ecosystem services OR biodiversity parameters of interest	O
Review design/methods Review, meta-analyses	Included: review with qualitative summary and/or meta-analyses. The review summarizes empirical studies Excluded: not a review article summarizing primary research	D

The ID column refers to the capital letter that will be used in the csv file to provide reasons for review exclusion. The number of studies excluded will also be displayed in the flow diagram (Additional file 3)

After every hierarchical level of the screening process, the two reviewers discuss their differences in decisions and resolve them together. To reduce the risk of missing eligible studies, in the case of ambiguity of the usefulness of the respective review, the review is always transferred to the next hierarchical level of the screening process. If the inclusion or exclusion of a certain review remains unclear at the full-text stage, the two reviewers discuss in detail the respective review and document their final reasoning for inclusion or exclusion. The screening process will be documented with a flow diagram [22, 52] for each of the four categories (Additional file 3).

To assess agreement between reviewers and test if the eligibility criteria are applied consistently, the alignment is checked with a kappa value [53] based on the first 100 screened reviews at the title stage of all four categories (for more details on the kappa value see Additional File 6). If there is at least a substantial agreement between reviewers (agreement on 81 of the 100 reviews, kappa > 0.6; [54]), the entire screening process will be conducted as planned. If there is less than substantial agreement, the reviewers discuss the eligibility criteria and may modify for clarification.

A full list of all reviews retrieved by the original search (duplicates removed) will be made available as a CSV file together with review details (author(s), document title, year, DOI, abstract, author keywords and indexed keywords). A list of reviews that remain after title and abstract screening, respectively, will also be published together with the final publication. The list of reviews at full-text stage will include an additional column indicating the reason for exclusion. The CSV files will be permanently made available with the research management tool by the Center for Open Science (Open Science Framework, https://osf.io/) according to the guidelines of open science [55] to support a transparent and repeatable screening process.

### Review validity assessment

Despite good and thorough guidelines for conducting evidence syntheses [22, 52], the term systematic review is erroneously used for traditional, narrative literature reviews, which are in most cases not conducted on a priori defined systematic methodology and therefore much more susceptible to biases [56]. Thus, the assessment of review validity deserves central attention when conducting a systematic map of reviews [57]. The validity of every review included in the summary table of scope (see section “Data synthesis and presentation”) will be assessed using a checklist based on the newest version of the Evidence Synthesis Appraisal Tool (CEESAT) by the Collaboration for Environmental Evidence (2022, Version 2.1, CEESAT for Evidence Reviews, can be downloaded at https://environmentalevidence.org/ceeder/about-ceesat/) by two independent reviewers. In addition to the five selected CEESAT criteria included in the summary of scope table of the final publication (Table 4), the full assessment of all 16 CEESAT criteria for every included review is provided as supplementary material (Additional file 4). Disagreements between the assessments of the two reviewers will be discussed and if there is no agreement whether or not a criterion is met by the review, both categories are displayed in the respective table to emphasize the disagreement of reviewers. In case the respective review included in this systematic map has already been assessed by the network of the CEEDER data base (https://environmentalevidence.org/ceeder/), the available assessment from the data base will be used and no additional assessment will be conducted.

**Table 4:** Summary table of scope of included reviews and assessments of selected CEESAT categories

Review year	RI^1^	Aim primary objective	No. of stud	Region^2^	DA^3^	protocol? (2.1)^4^	Search comprehensive (3.2)^4^	Eligibility criteria defined (4.1)^4^	Critical appraisal (5.1)^4^	Limitations discussed (8.1)^4^
Name1and Name2 (2012) ^5^	1	Investigate the effect of salvage logging on tree regeneration	21	Global						
Name 3 et al. 2019 ^5^	a	…								

The final publication will also provide a table with the full CEESAT assessments for every included review and a table template is provided here as Additional file 4

^1^Review Identifier; number: reference with meta-analysis (quantitative synthesis), letter: reference with narrative synthesis (qualitative synthesis) following[30]

^2^Global, Tropical, Temperate, Boreal

^3^Disturbance Agent: all, drought, fire, wind, ice and snow, pathogens, insects, herbivores, harvest

^4^based on CEESAT. Number in brackets refers to the section in CEESAT (2020, Version 2.1): Is there an a-priori method/protocol document? (2.1); Is the search comprehensive? (3.2); Are eligibility criteria clearly defined? (4.1); Does the review critically appraise each study? (5.1); Have the authors considered limitations of the synthesis? (8.1)

^5^article access issues: open book indicates open access and closed book indicates some form of subscription

**Fig. 2:**
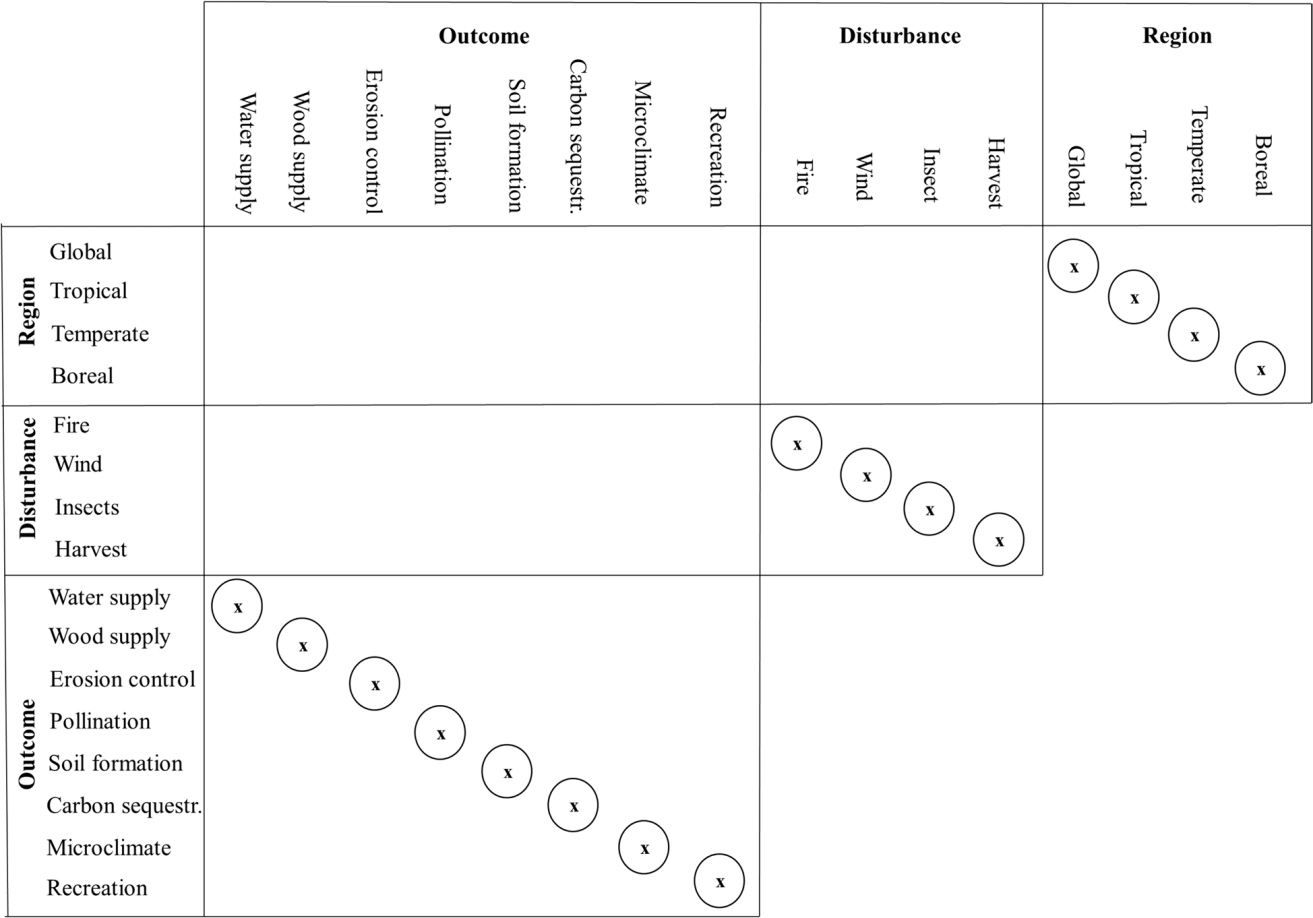
Matrix summarizing the total number of reviews considering each question with respect to ecosystem services. The matrix dimensions are based on the key characteristic outlined with Fig. 1. The matrix can be read using combinations from the left and top headings to form the question of interest, e.g. “What is the number of reviews that were conducted for the temperate region that considered the outcome pollination?”. The diagonals indicate the total number of reviews conducted for the respective category. For further explanation regarding this visualization see [30]

**Fig. 3:**
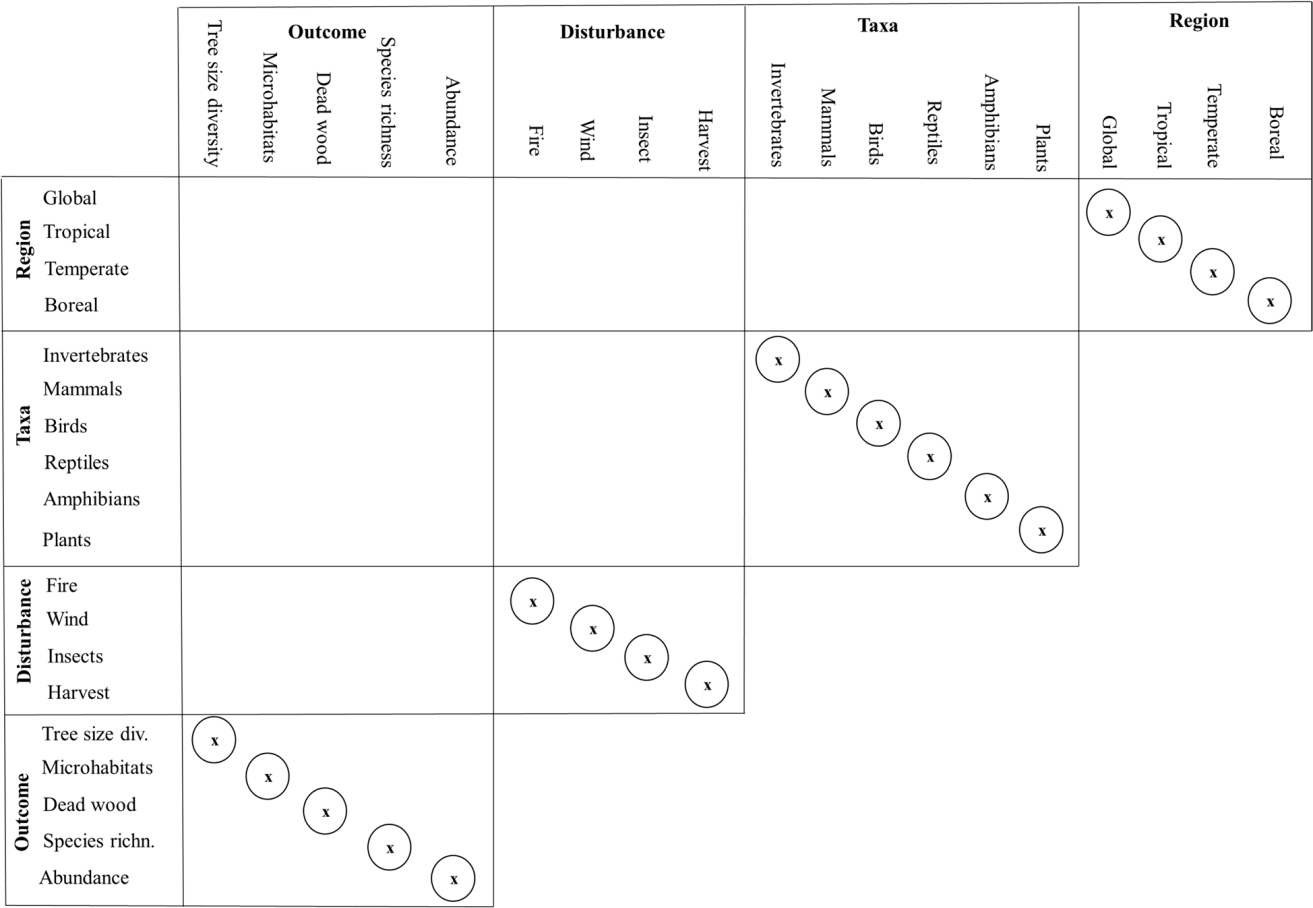
Matrix summarizing the total number of reviews considering each question with respect to biodiversity. The matrix dimensions are based on the key characteristic outlined with Fig. 1. The matrix can be read using combinations from the left and top headings to form the question of interest, e.g. “What is the number of reviews that were conducted for the disturbance agent fire that considered the taxa of reptiles?”. The circled diagonals indicate the total number of reviews conducted for the respective category. For further explanation regarding this visualization see [30]

Despite the convenience to rate the overall confidence of the included reviews and a method for scoring an evidence synthesis was provided by the original publication of CEESAT [58], we do not plan to provide the reader with an overall score. It would prevent the reader from carefully considering the individual review and checking which of the CEESAT criteria are more important for their environmental management question. To provide scores may seem desirable for a systematic map of reviews to estimate the overall risk of bias and the overall confidence in the results. It disguises, however, the potential relevance of a review for a reader behind a single number. Nevertheless, if a review has a majority of amber and red CEESAT assessments (deficient and seriously deficient in conduct and/or reporting) the confidence in the finding can be expected to be critically low. Therefore, if 13 or more CEESAT criteria are graded amber/red (over 80%), the respective review is excluded from the summary of findings table (Tables 5 and 6) and not visualized in the matrix (Figs. 2 and 3) but only listed in the summary of scope table (see flow diagram Additional file 3). A review is also immediately excluded from the findings table if the choice of synthesis approach is inappropriate and graded red (e.g., the review uses vote-counting: “most studies found”, “three out of ten indicated” [59], see also Problem 7 Inappropriate synthesis in [60]).

**Table 5:** Summary of findings table of individual outcomes for Ecosystem services

Region	Ecosystem service section	Outcome measure	review year	RI^1^	DA^2^	Outcome indicator	Results effect size + standard error	Key findings^3^
Global	Provisioning	Water supply	Name4 et al. (2011)	5	wind			
			Name5 (2021)	c	fire			
		Wood supply	Name1and Name2 (2012)	1	all	Tree density and height	height: -0.19 + 0.17 Density: −0.18 + 0.3	No evidence for a general effect of salvage logging on tree density and regeneration
	Regulating	Erosion control						
		Pollination						
		Soil formation						
		Carbon sequestration						
		Microclimate						
	Cultural	Recreation						
Tropical	Provisioning	Water supply						
		Wood supply						
	Regulating	Erosion control						
		Pollination						
		Soil formation						
		Carbon sequestration						
		Microclimate						
	Cultural	Recreation						
Temperate	Provisioning	Water supply						
		Wood supply						
	Regulating	Erosion control						
		Pollination						
		Soil formation						
		Carbon sequestration						
		Microclimate						
	Cultural	Recreation						
Boreal	Provisioning	Water supply						
		Wood supply						
	Regulating	Erosion control						
		Pollination						
		Soil formation						
		Carbon sequestration						
		Microclimate						
	Cultural	Recreation						

Language of evidence [62] is used to describe the effect to avoid the use of the term “statistically significant” (for a discussion see for example [63–65])

^1^Review identifier, see summary table of scope (Table X)

^2^Disturbance agent: all, fire, wind, insects, harvest

^3^Language of evidence

**Table 6:** Summary of findings table of individual outcomes for Biodiversity

	Ecosystem service type	Outcome measure	Review Year	RI^1^	DA^2^	Taxa^3^	Outcome measures	Results Effect size + standard error	Key findings^4^
Global	Structure	Microhabitats	Name4 et al. (2011)						
			Name5 (2021)						
		Dead wood							
		Tree size div							
	Composition	Species richn							
		Abundance							
Tropical	Structure	Microhabitats							
		Dead wood							
		Tree size div							
	Composition	Species richn							
		Abundance							
Temperate	Structure	Microhabitats							
		Dead wood							
		Tree size div							
	Composition	Species rich							
		Abundance							
Boreal	Structure	Microhabitats							
		Dead wood							
		Tree size div							
	Composition	Species rich							
		Abundance							

Language of evidence [62] is used to describe the effect to avoid the use of the term “statistically significant” (for a discussion see for example [63–65])

^1^Review identifier, see summary table of scope (Table X)

^2^Disturbance Agent: all, fire, wind, insects, harvest

^3^Taxa: All (all), Plants (P), Invertebrates (I), Mammals (M), Birds (B), Reptiles (R), Amphibians (A)

^4^Language of evidence [62] is used to describe the effect; avoidance of the term “statistically significant” (for a discussion see for example [63–65]))

### Data coding strategy

There will be no data extraction from primary studies, and all the gathered information originates from quantitative and qualitative data summarized in the reviews. Table templates for each of the four forest management questions are provided to systematically extract data for every eligible review in the same way (Tables 4, 5, and 6). Two review authors will extract the data from the reviews independently. After the independent extraction process, the content of every table entry is directly compared, differences discussed and resolved together.

There is no risk that the persons planned to extract the data has (co-)authored one of the reviews included.

### Review mapping and presentation

The systematic map will include the results of all eligible reviews and tabulate them in a summary of scope and summary of findings table [26]. The systematic map will include one summary table of scope of reviews (Table 4) and one summary of findings table per forest management question (Tables 5 and 6). The summary of findings table is subdivided into the different outcome types for ecosystem services (Table 5) and biodiversity (Table 6).

We expect that more than one review exists to investigate the effects of one of the interventions, and the likely overlap in primary research studies included in different reviews needs to be recognized as a limitation. Assessing the degree of overlap between evidence syntheses is anticipated to be a complex and time-consuming task [26]. Therefore, there is no aggregation of effect sizes from different reviews and the synthesis in this systematic map can be classified as an “evidence review map” [30]. To complement the tables we visualize the scope of our systematic map as a matrix that locates the number of reviews within our covered review landscape (Figs. 2 and 3 following [30, 40]).

In line with the PRIOR items 19b and 19c [61], we will also provide a table as supplementary material that presents the results of testing for causes of heterogeneity and if a meta-analysis was conducted, it shortly presents the results of a sensitivity analysis (Additional file 2). For this purpose, we use the information and results presented in the respective review and do not carry out any further analyses ourselves.

## Supplementary Information

**Supplementary material 1.** ROSES form for systematic map protocols.

**Supplementary material 2.** Assessment of meta-analysis results of included studies.

**Supplementary material 3.** Flow Diagram.

**Supplementary material 4.** CEESAT categories and full CEESAT assessment table template.

**Supplementary material 5.** Test of comprehensiveness of the search results.

**Supplementary material 6.** Cohens Kappa Reviewer Agreement.

**Supplementary material 7.** Search Strings Web of Science and CABI Abstracts Forest Science Library.

## Data Availability

No data were generated for this systematic map protocol. All relevant materials are included in this published protocol and its additional files.
