# Supplementary material for: What secondary research evidence exists on the effects of forest management after disturbances: a systematic map protocol
Source: Environ Evid. 2024 Jun 2;13:16. doi: 10.1186/s13750-024-00340-7 (PMC11378863; doi:10.1186/s13750-024-00340-7)
Supplement: Supplementary file 1 — Supplementary material 1. ROSES form for systematic map protocols. [file 13750_2024_340_MOESM1_ESM.pdf]

number	Section / sub-section	Topic	Description	Further explanation	Checklist/Meta-data	Author response	Comments
1	Title	Title	indicate if it is an update/amendment: e.g. "A systematic map update	question.	Meta-data	overview of reviews	
2	Type of review	Type of review	map update, systematic map amendment	updates [2]	Meta-data	systematic map	
3	Authors contacts	Authors contacts	authors must be provided.		Checklist	Yes	
4	Abstract	Structured summary	Background, the context and purpose of the review, including the review		Checklist	Yes	
5	Background	Background	known. Protocol must indicate why this study was necessary and what it	the intervention or exposure to the outcome.	Checklist	Yes	
6	Stakeholder engagement	Stakeholder engagement	(e.g. in the formulation of the question) must be described and explained		Checklist	Yes	
7	Objective of the review	Objective	applicable).	questions are usually linked to sources of heterogeneity (effect	Checklist	Yes	
8		components	intervention(s)/exposure(s), comparator(s), and outcome(s).	For other question types see [4,5]	Meta-data	PICO	
	Methods						
9	Searches	Search strategy		Details regarding search strategy testing should be provided.	Checklist	No	
10		Search string	the string is formatted (e.g. Web of Science format)		Meta-data	Yes	Scopus format
11		databases	List languages to be used in bibliographic database searches.		Meta-data	Yes, english	English
12		Languages – grey literature	based search engines.		Meta-data	No	No search for grey literature
13		Bibliographic databases	Provide the number of bibliographic databases to be searched.		Meta-data	3	
14		Web – based search engines	Provide the number of web – based search engines to be searched.		Meta-data	0	
15		Organisational websites	Provide the number of organisational websites to be searched.		Meta-data	0	
16		comprehensiveness of the search	strategy was assessed (i.e. list of benchmark articles).		Checklist	Yes	
17		Search update	review.	performed more than two years prior to review completion.	Checklist	No	
18	inclusion criteria	Screening strategy	relevance/eligibility.		Checklist	Yes	
19		Consistency checking	including the levels at which consistency checking will be undertaken and		Checklist	Yes	
20		Inclusion criteria	articles/studies. These must be broken down into the question key		Checklist	Yes	
21		Reasons for exclusion	reasons for exclusion.		Checklist	Yes	
22	Critical appraisal	Critical appraisal strategy	validity (including assessment of individual studies and the evidence base	Optional	Checklist	Yes	CEESAT
23		Critical appraisal used in synthesis	synthesis.	Optional	Checklist	Yes	
24		Consistency checking	tested.	Optional	Checklist	Yes	
25	Data extraction	strategy	(potentially providing forms/data sheets (ideally piloted), list if variables		Checklist	Yes	
26	presentation	Narrative synthesis strategy	base in the form of descriptive statistics, tables (including SM database)	of their findings) must be avoided. May include a summary of the outputs	Checklist	Yes	
27		identification strategy	knowledge gaps (unrepresented or underrepresented subtopics that		Checklist	Yes	
28		independence	articles to be considered within the review) in decisions regarding	should be prevented from unduly influencing inclusion decisions, for	Checklist	Yes	
29	Declarations	Competing interests	review authors may have.		Checklist	Yes	

References

[1] James, K.L., Randall, N.P. and Haddaway, N.R., 2016. A methodology for systematic mapping in environmental sciences. Environmental Evidence, 5(1), p.7.
[2] Bayliss, H.R., Haddaway, N.R., Eales, J., Frampton, G.K. and James, K.L., 2016. Updating and amending systematic reviews and systematic maps in environmental management. Environmental Evidence, 5(1), p.20.
[3] Haddaway, N.R., Kohl, C., da Silva, N.R., Schiemann, J., Spök, A., Stewart, R., Sweet, J.B. and Wilhelm, R., 2017. A framework for stakeholder engagement during systematic reviews and maps in environmental management. *Environmental Evidence* , *6* (1), p.11.
[4] Collaboration for Environmental Evidence. 2018. Guidelines and Standards for Evidence synthesis in Environmental Management. Version 5.0. www.environmentalevidence.org/information-for-authors.
[5] Leeds Institute of Health Sciences. https://medhealth.leeds.ac.uk/info/639/information_specialists/1500/search_concept_tools. Accessed 12/11/2017.
