# Supplementary material for: What secondary research evidence exists on the effects of forest management after disturbances: a systematic map protocol
Source: Environ Evid. 2024 Jun 2;13:16. doi: 10.1186/s13750-024-00340-7 (PMC11378863; doi:10.1186/s13750-024-00340-7)
Supplement: Supplementary file 2 — Supplementary material 2. Assessment of meta-analysis results of included studies. [file 13750_2024_340_MOESM2_ESM.docx]

Supplementary Material

**What secondary research evidence exists on the effects of forest management after disturbances: a Systematic Map Protocol**

Moritz Baumeister, Markus Meyer

**Additional file 2: Assessment of meta-analyses results of included studies.**

**Table A2.1** Results of investigations of potential causes of heterogeneity and sensitivity analyses of conducted meta-analyses (following PRIOR items 19b and 19c, Gates *et al.* 2022)

| **Review**  **Year** | **RI^1^** | **Effect modifiers tested** | **Results** | **SA^2^** | **Results** |
| --- | --- | --- | --- | --- | --- |
|  |  | e.g. geographical location, mean age of forest stand |  | **yes** | e.g. The results were sensitive to different levels of study inclusion criteria. |
|  |  |  |  |  |  |
|  |  |  |  |  |  |
| ^1^ Review Identifier; number: reference with meta-analysis (quantitative synthesis), letter: reference with narrative synthesis (qualitative synthesis) following O’Leary *et al.* 2017.  ^2^ Sensitivity analysis conducted? Yes or no | | | | | |
