# Supplementary material for: What secondary research evidence exists on the effects of forest management after disturbances: a systematic map protocol
Source: Environ Evid. 2024 Jun 2;13:16. doi: 10.1186/s13750-024-00340-7 (PMC11378863; doi:10.1186/s13750-024-00340-7)
Supplement: Supplementary file 3 — Supplementary material 3. Flow Diagram. [file 13750_2024_340_MOESM3_ESM.pptx]

## Slide 1
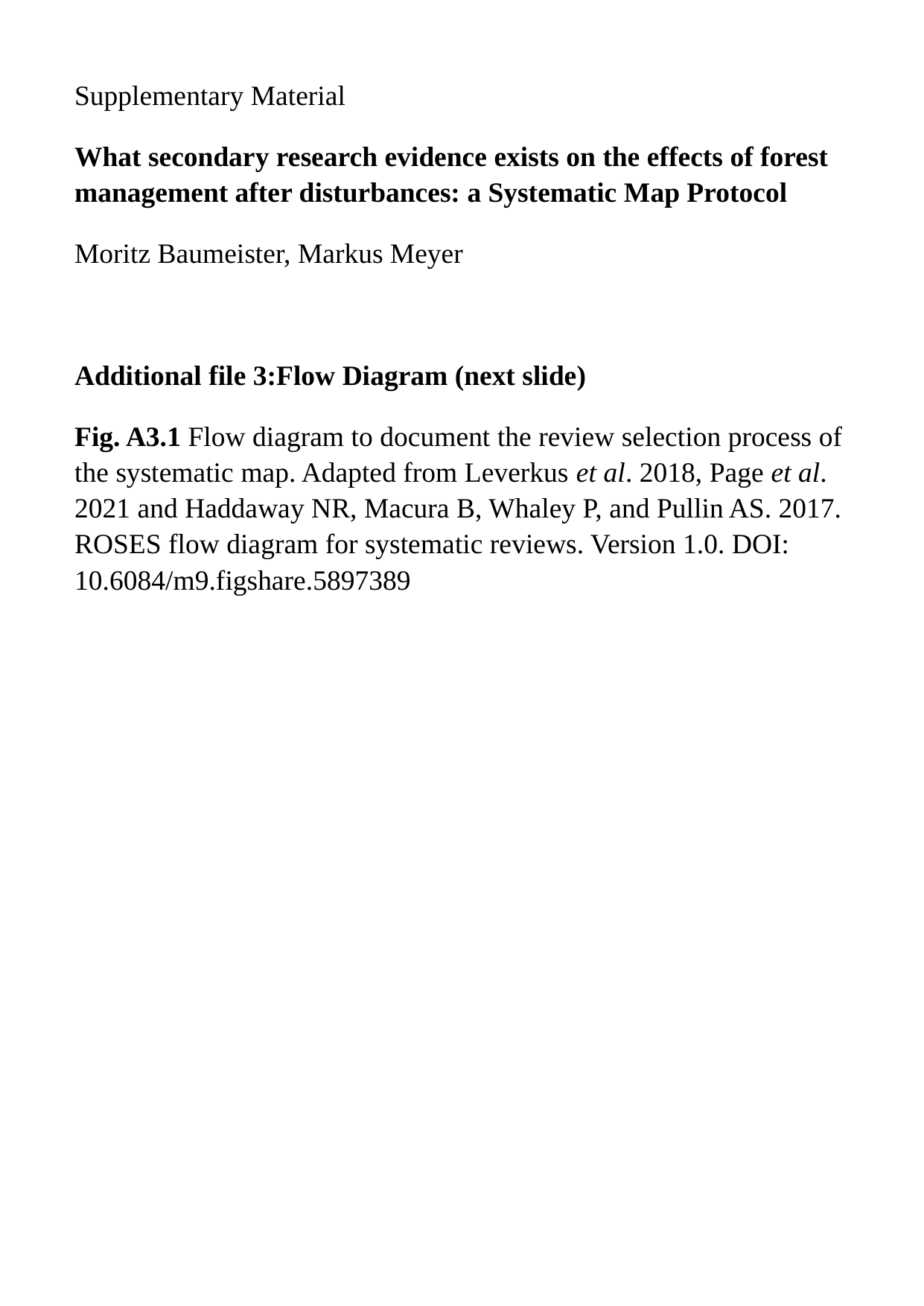

Supplementary Material
What secondary research evidence exists on the effects of forest management after disturbances: a Systematic Map Protocol
Moritz Baumeister, Markus Meyer
Additional file 3:Flow Diagram (next slide)
Fig. A3.1 Flow diagram to document the review selection process of the systematic map. Adapted from Leverkus et al. 2018, Page et al. 2021 and Haddaway NR, Macura B, Whaley P, and Pullin AS. 2017. ROSES flow diagram for systematic reviews. Version 1.0. DOI: 10.6084/m9.figshare.5897389

## Slide 2
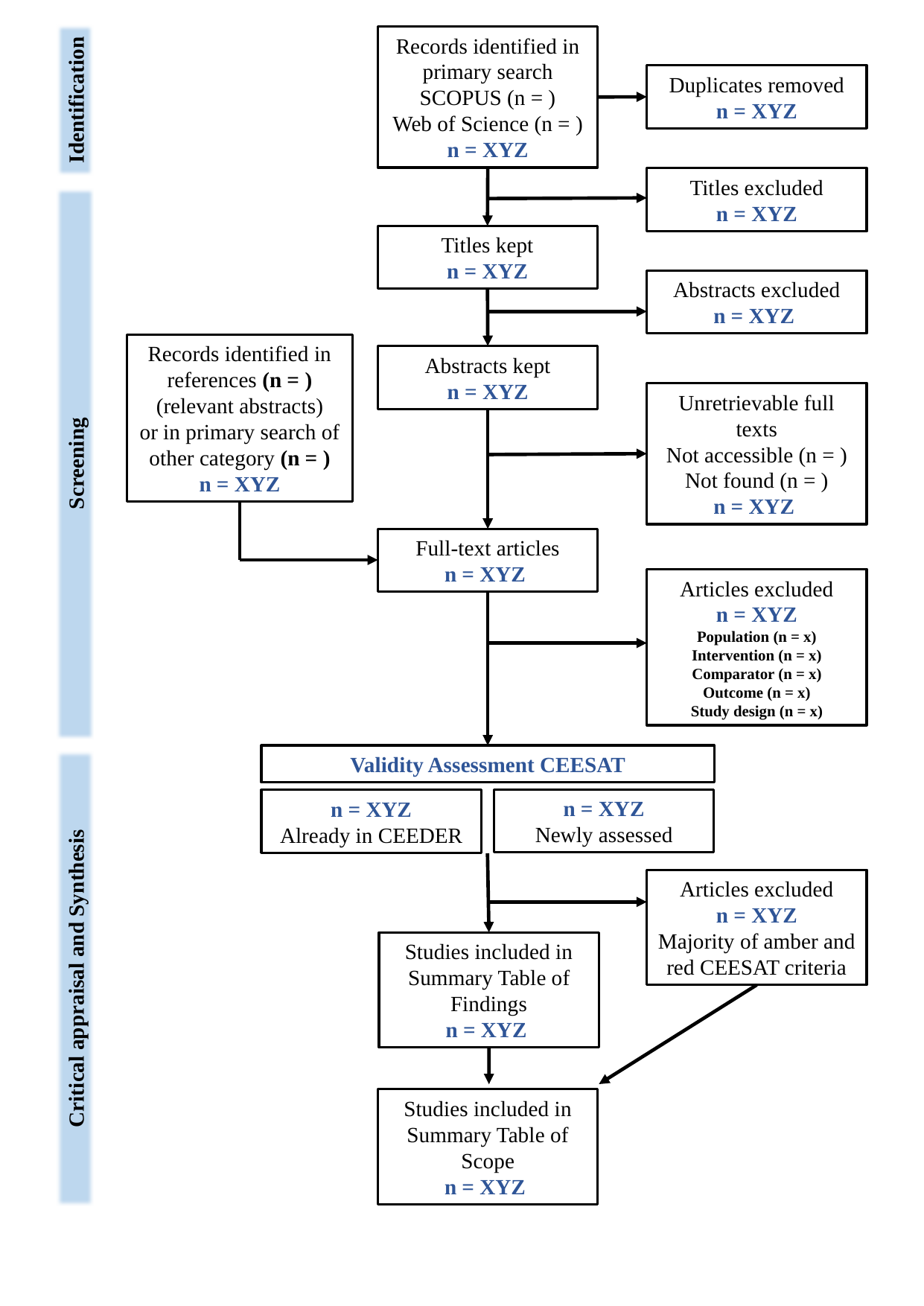

Records identified in primary search SCOPUS (n = )
Web of Science (n = )
n = XYZ
Duplicates removed
n = XYZ
Identification
Titles excluded
n = XYZ
Titles kept
n = XYZ
Abstracts excluded
n = XYZ
Records identified in references (n = ) (relevant abstracts)
or in primary search of other category (n = )
n = XYZ
Abstracts kept
n = XYZ
Unretrievable full texts
Not accessible (n = )
Not found (n = )
n = XYZ
Screening
Full-text articles
n = XYZ
Articles excluded
n = XYZ
Population (n = x)
Intervention (n = x)
Comparator (n = x)
Outcome (n = x)
Study design (n = x)
Validity Assessment CEESAT
n = XYZ
Newly assessed
n = XYZ
Already in CEEDER
Articles excluded
n = XYZ
Majority of amber and red CEESAT criteria
Studies included in Summary Table of Findings
n = XYZ
Critical appraisal and Synthesis
Studies included in Summary Table of Scope
n = XYZ
