# Supplementary material for: What secondary research evidence exists on the effects of forest management after disturbances: a systematic map protocol
Source: Environ Evid. 2024 Jun 2;13:16. doi: 10.1186/s13750-024-00340-7 (PMC11378863; doi:10.1186/s13750-024-00340-7)
Supplement: Supplementary file 4 — Supplementary material 4. CEESAT categories and full CEESAT assessment table template. [file 13750_2024_340_MOESM4_ESM.docx]

Supplementary Material

**What secondary research evidence exists on the effects of forest management after disturbances: a Systematic Map Protocol**

Moritz Baumeister, Markus Meyer

**Additional file 4** CEESAT categories and full CEESAT Assessment

| **Table A4.1** Rating of 16 CEESAT criteria using four categories and their corresponding symbols. Description of categories was copied without modification from the CEE website (https://environmentalevidence.org/ceeder/about-ceesat/). From the same page you can also access the detailed descriptions of the four categories for every of the 16 criteria (Collaboration for Environmental Evidence 2020. The Collaboration for Environmental Evidence Synthesis Appraisal Tool (CEESAT). Version 2.1.) For basis of illustrations (CC 0; https://creativecommons.org/publicdomain/zero/1.0/) see cocomaterial.com | |  |
| --- | --- | --- |
| Gold: Meets the standards of conduct and/or reporting that reduce risk of bias as much as could reasonably be expected. Lowest risk of bias – high repeatability – highest reliability/confidence in findings. | 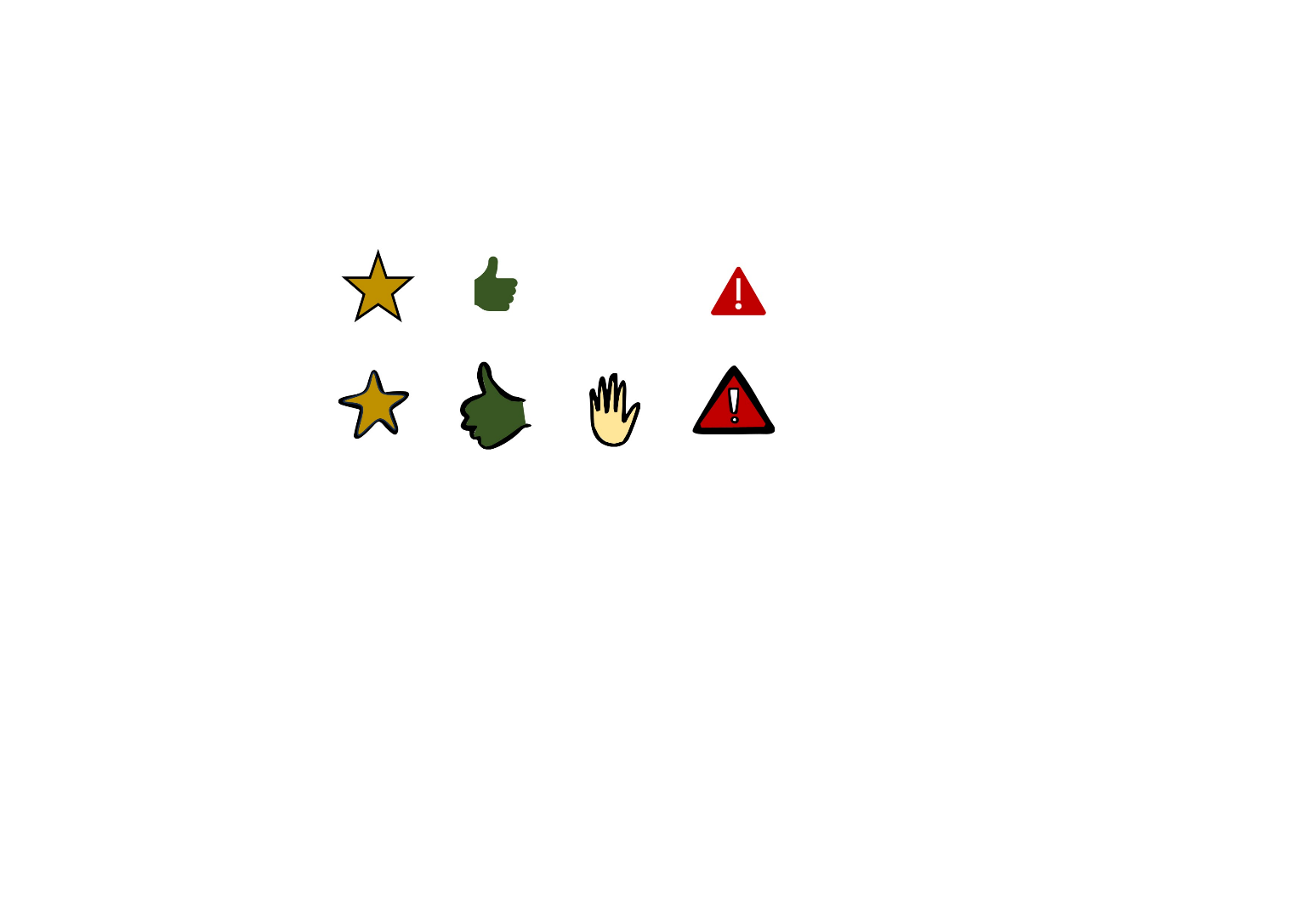 | |
| Green: Acceptable standard of conduct/reporting that reduces risk of bias. Acceptable risk of bias – repeatable – acceptable reliability/confidence in findings. | 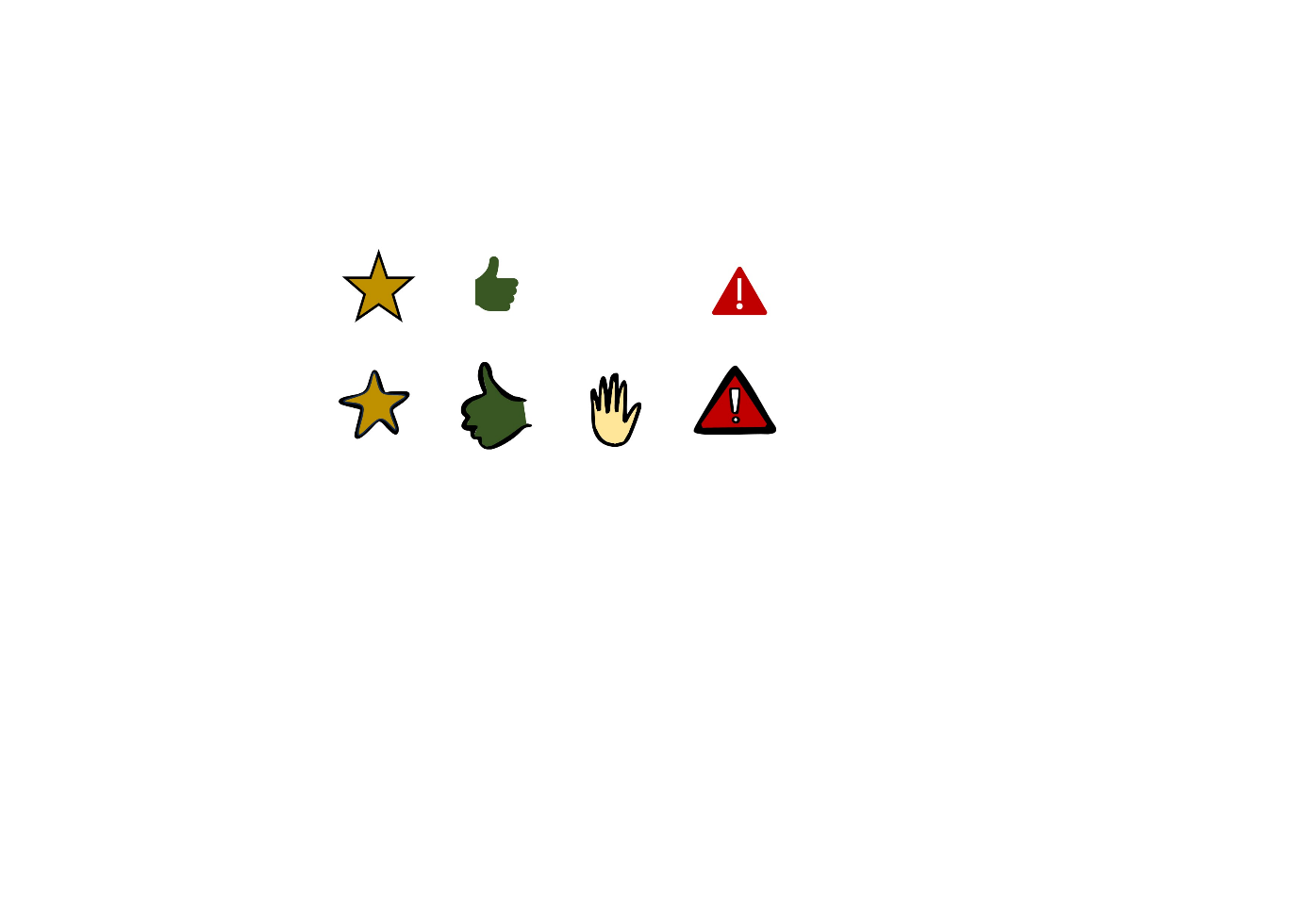 | |
| Amber: Deficiencies in conduct and/or reporting standards such that the risk of bias is increased (above green), alternatively risk of bias may be less easy to assess. Medium risk of bias – not fully repeatable – low reliability/confidence in findings | 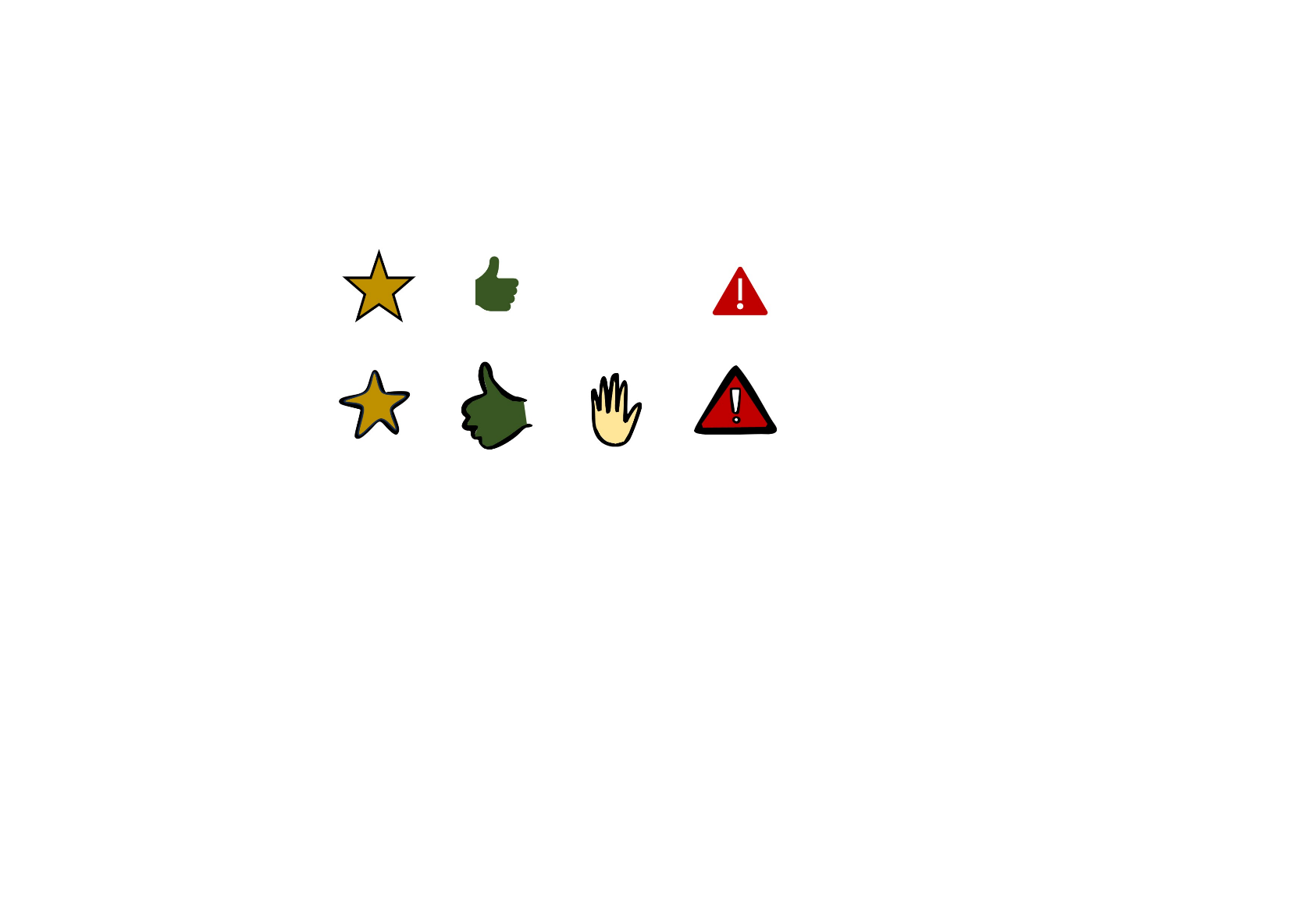 | |
| Red: Serious deficiencies in conduct and/or reporting such that risk of bias is high. High risk if bias – not repeatable – little to no confidence in findings | 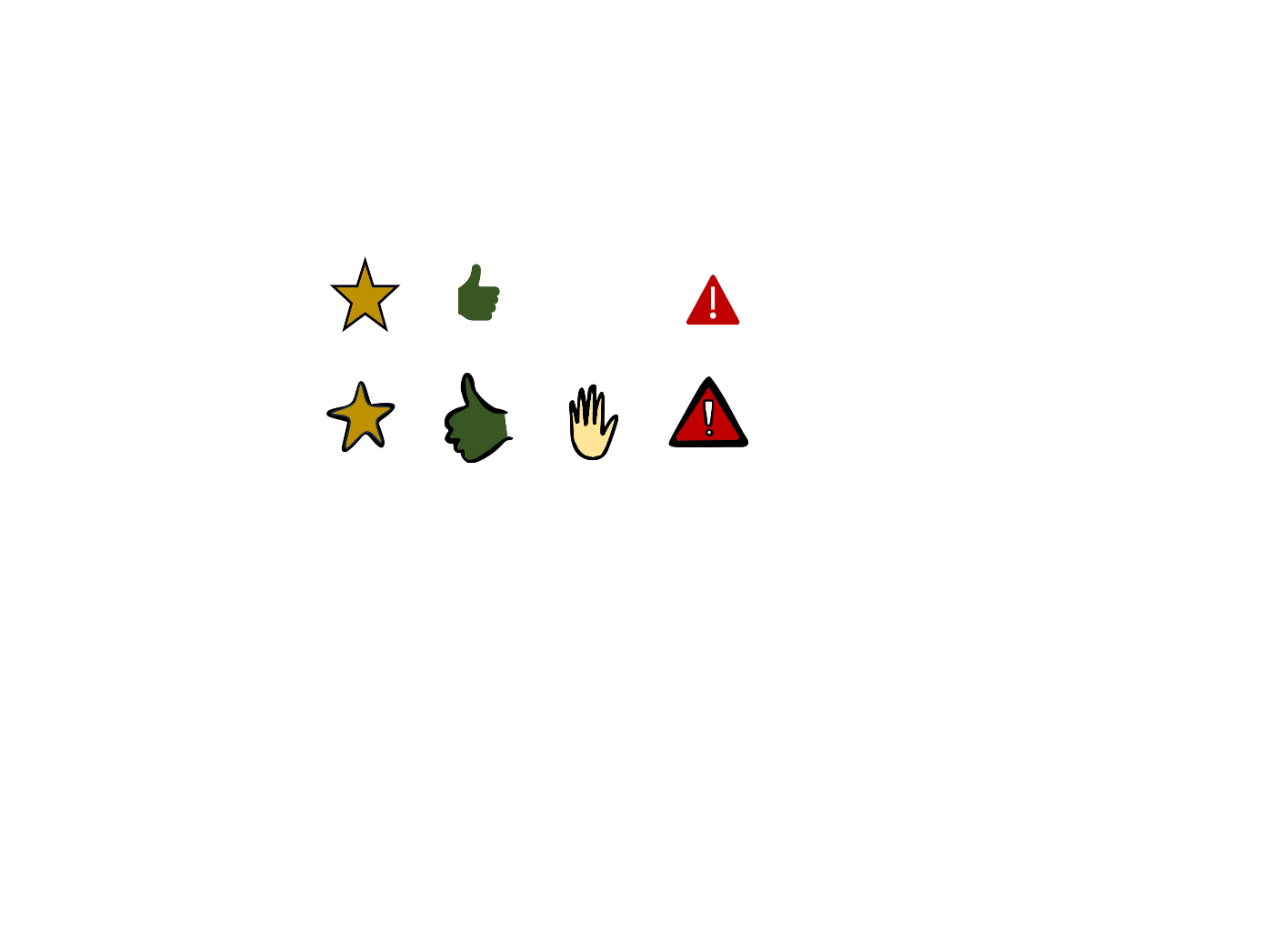 | |

| **Review**  **Year** | **RI^a^** | **CEEDER?^b^** | **1.1** | **2.1** | **3.1** | **3.2** | **4.1** | **4.2** | **4.3** | **5.1** | **5.2** | **6.1** | **6.2** | **6.3** | **7.1** | **7.2** | **7.3** | **8.1** | |
| --- | --- | --- | --- | --- | --- | --- | --- | --- | --- | --- | --- | --- | --- | --- | --- | --- | --- | --- | --- |
| **Name et al. 2008** | **5** | **no** | 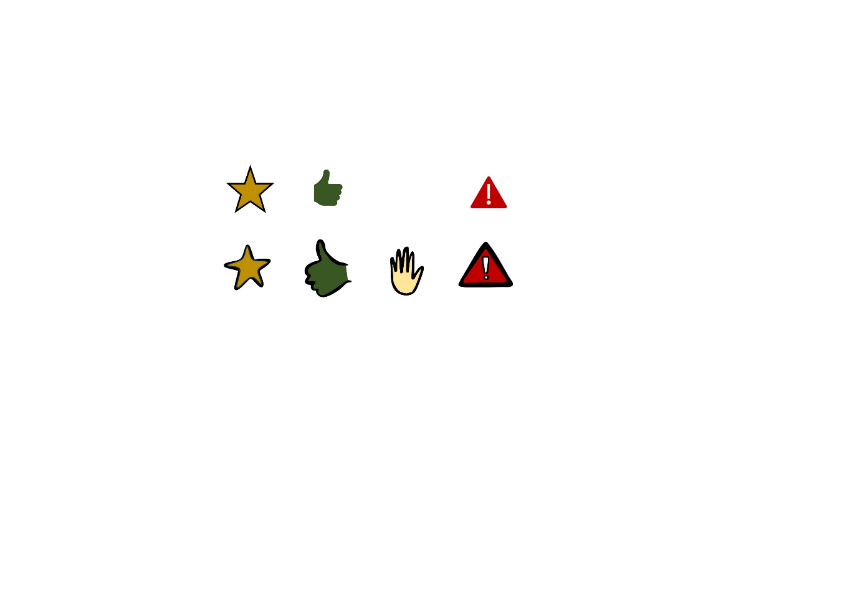 | 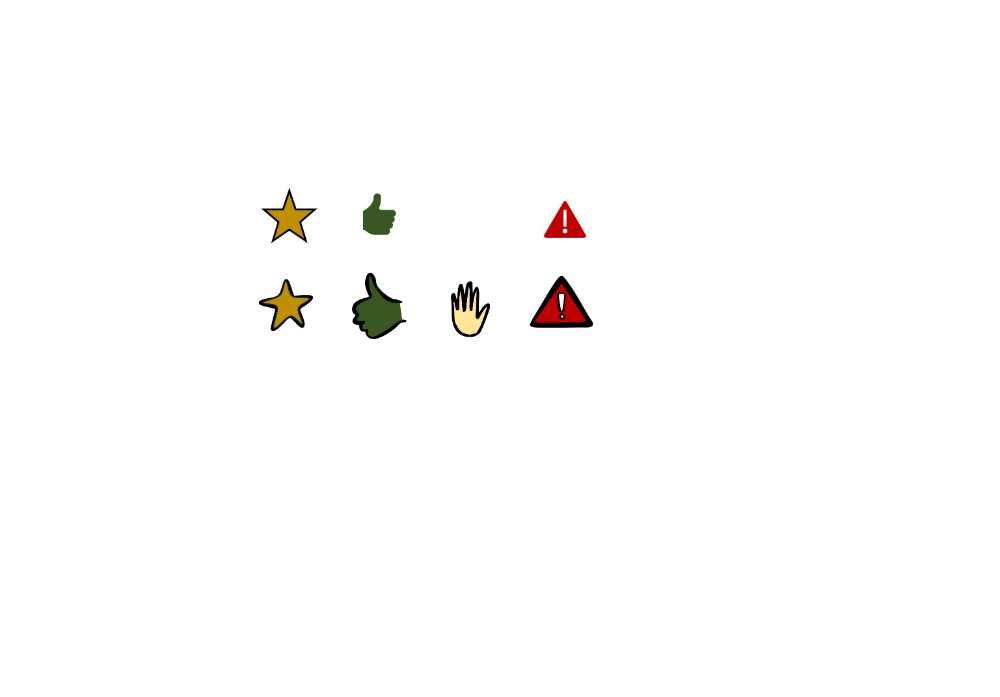 | 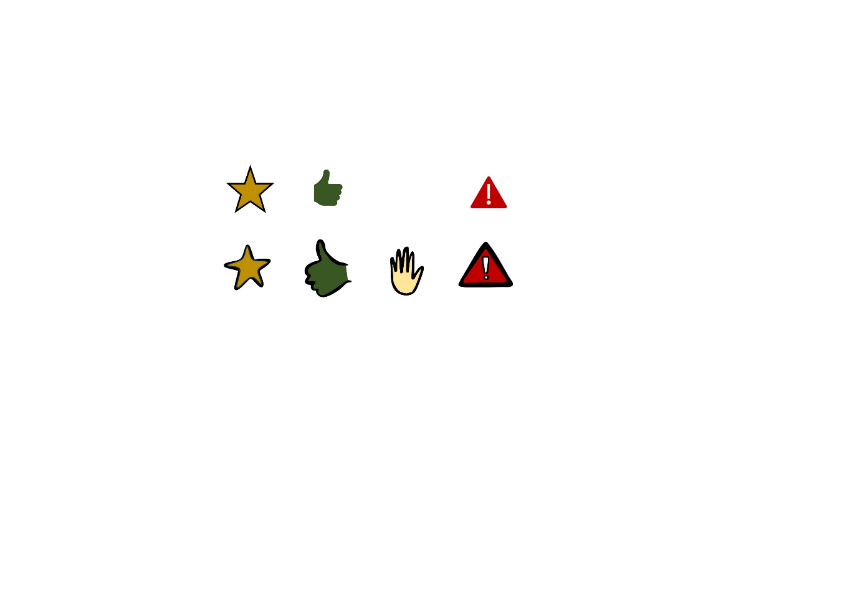 | 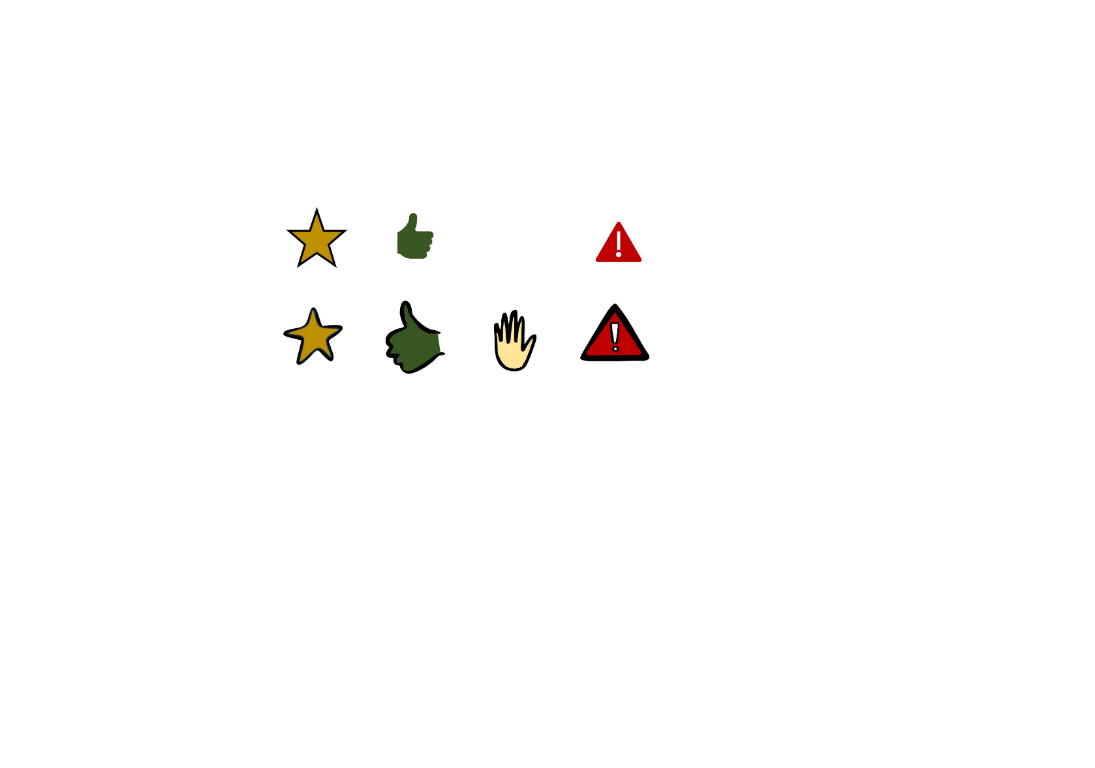 | 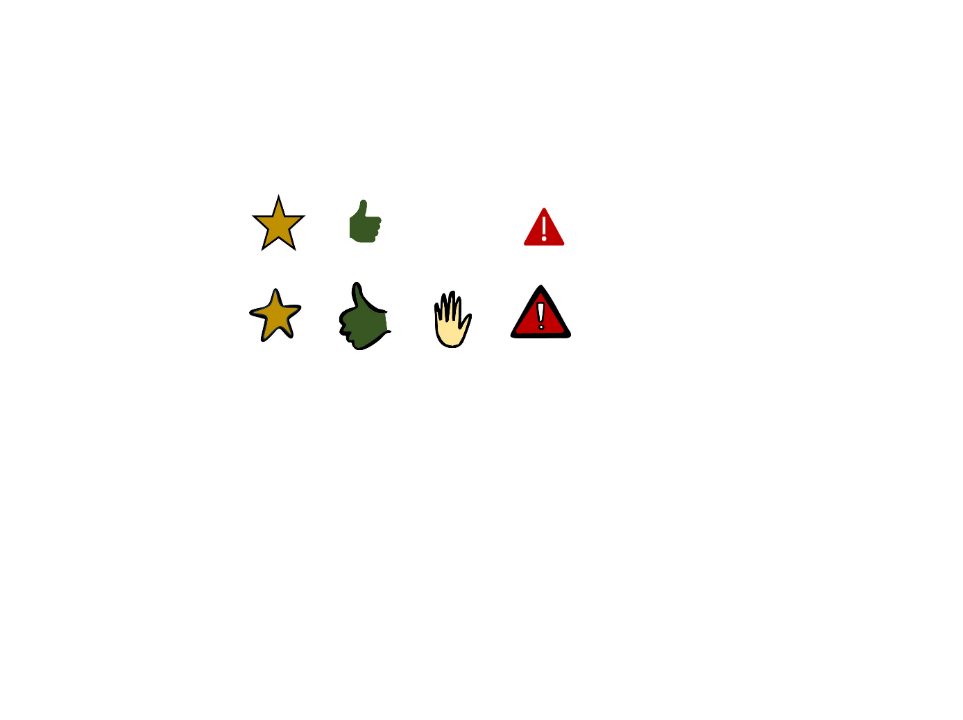 | 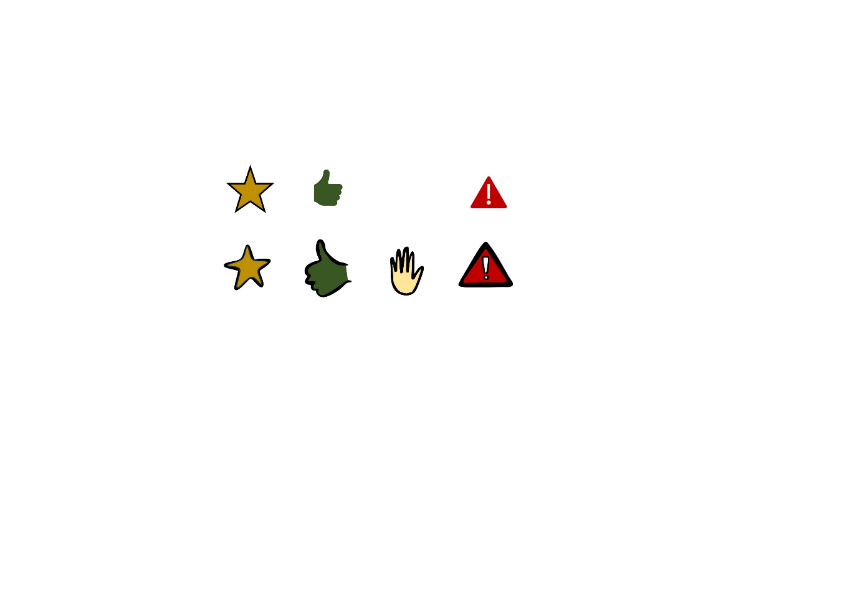 | 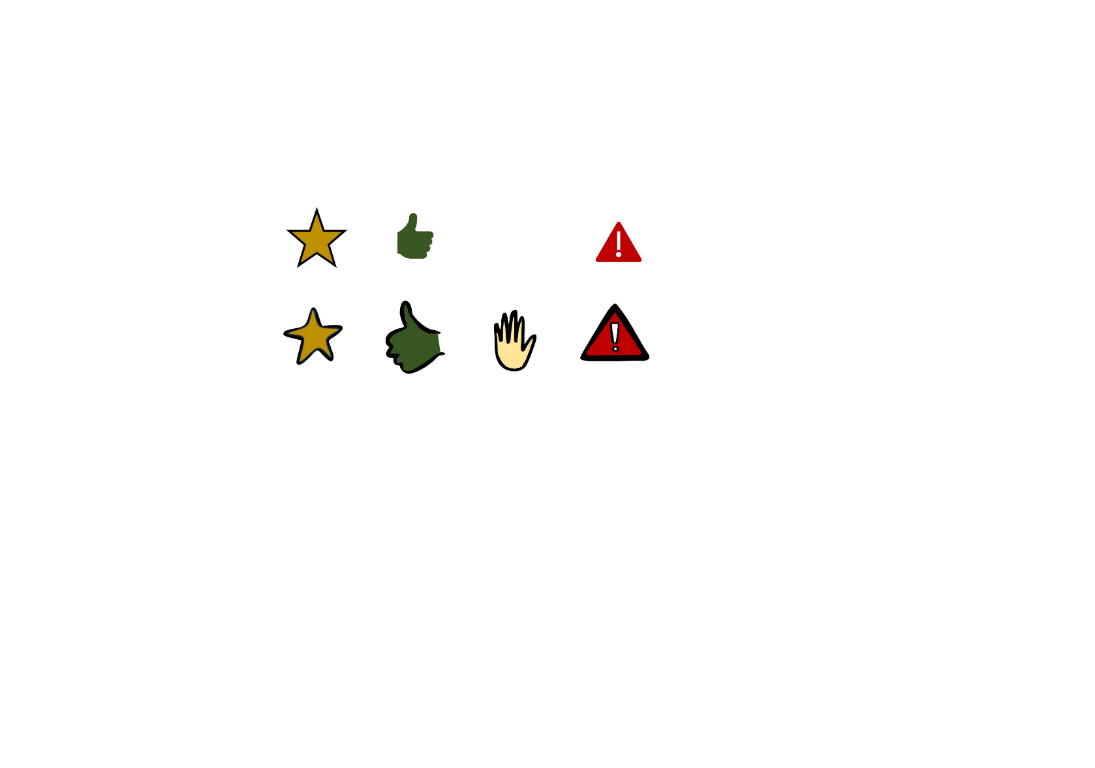 | 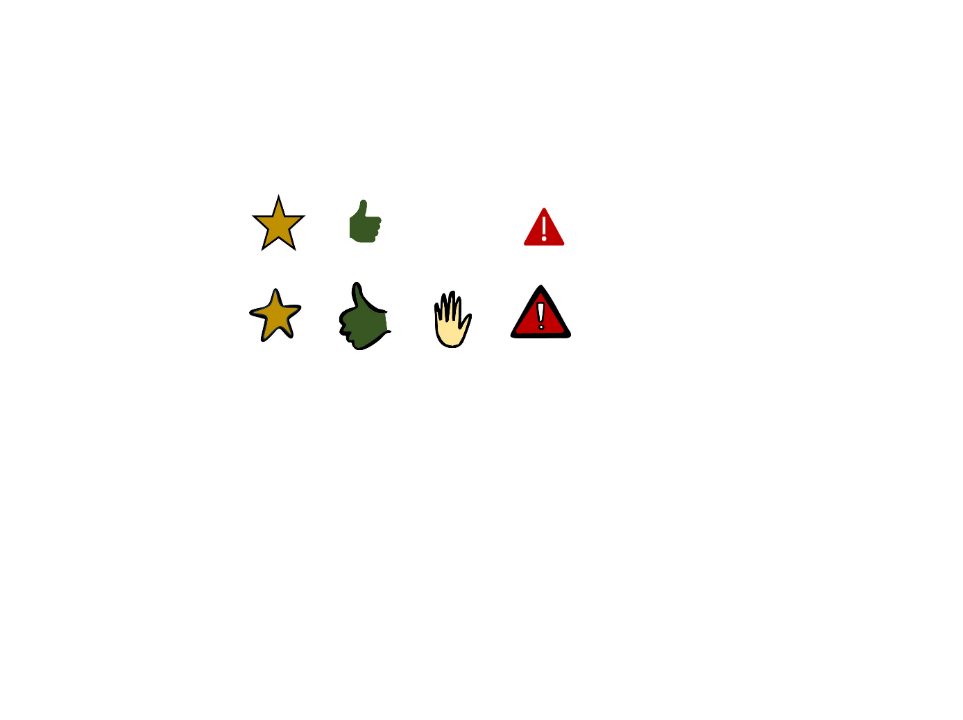 | 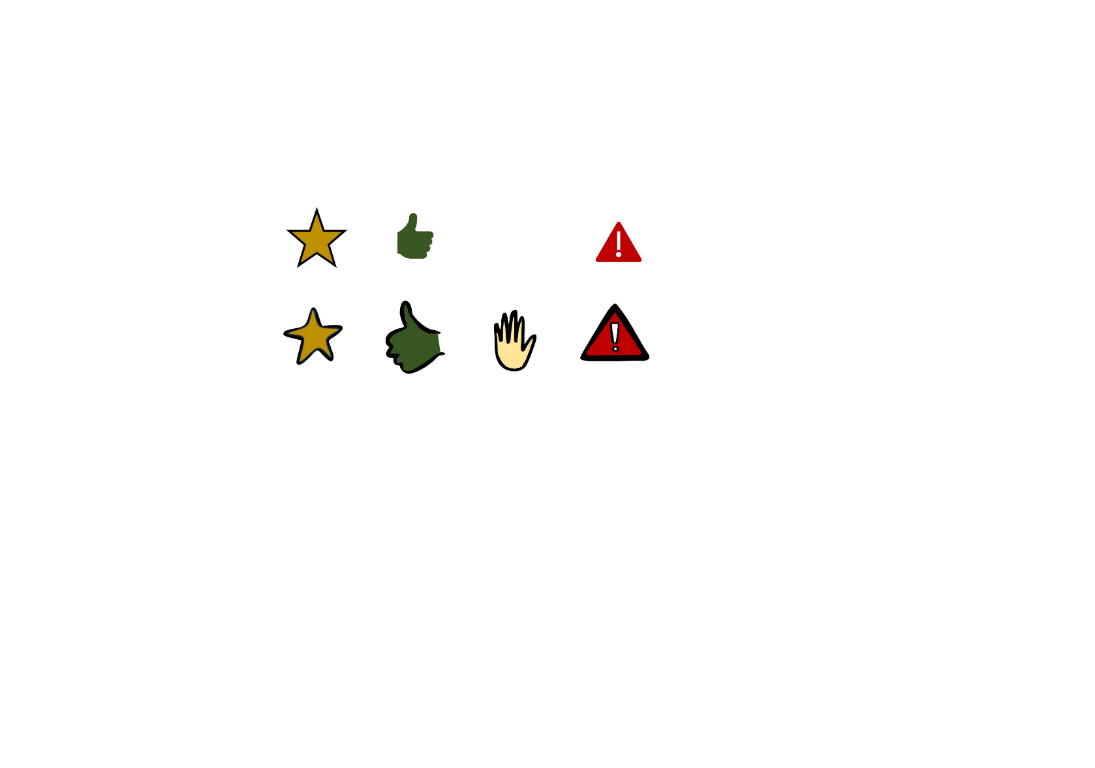 | 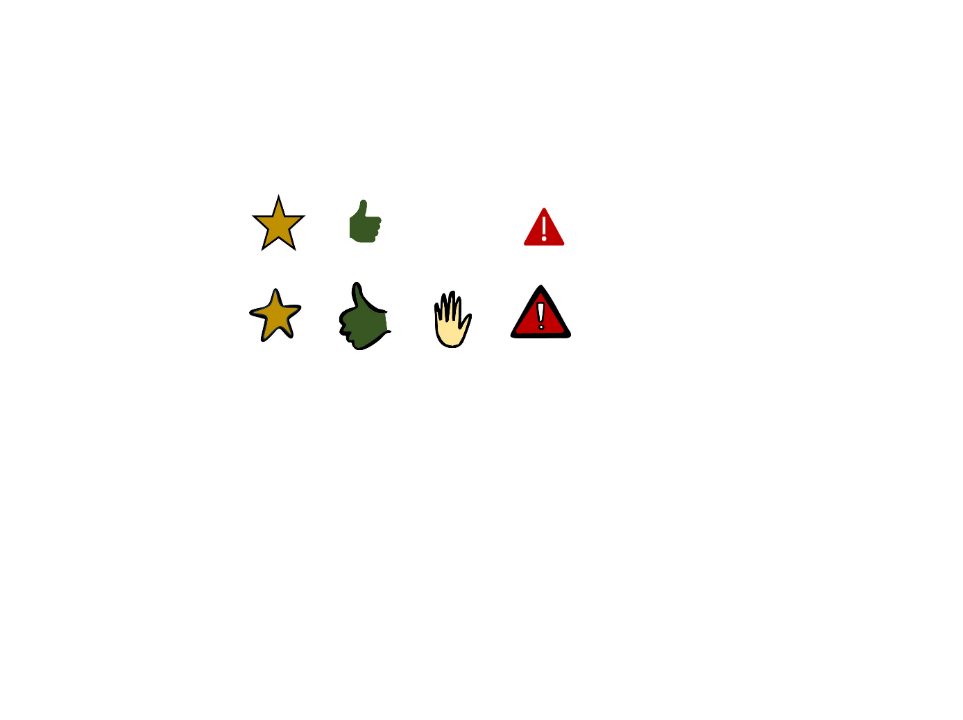 | 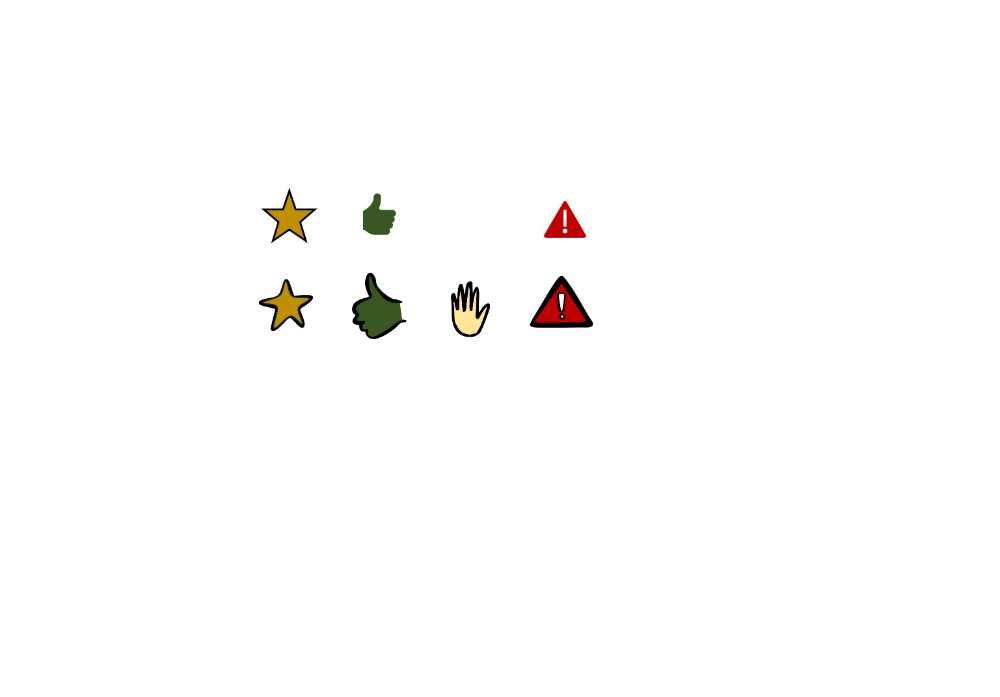 | 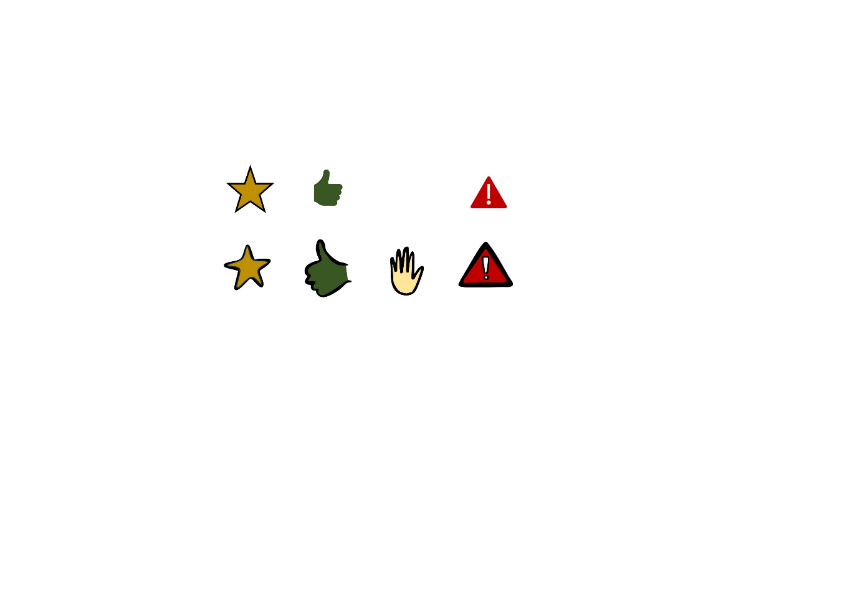 | 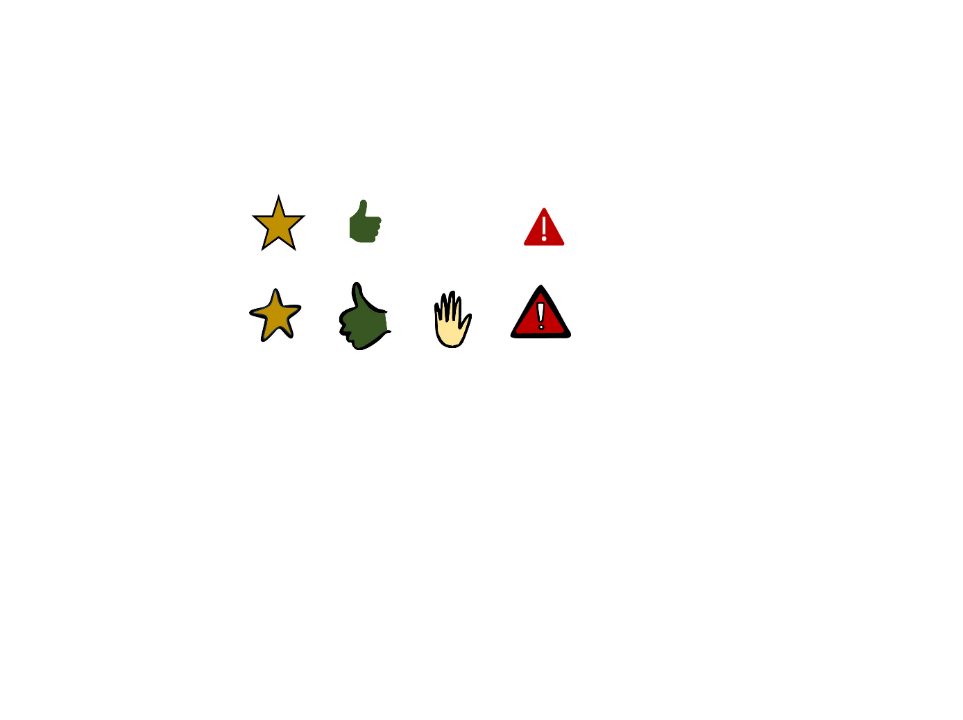 | 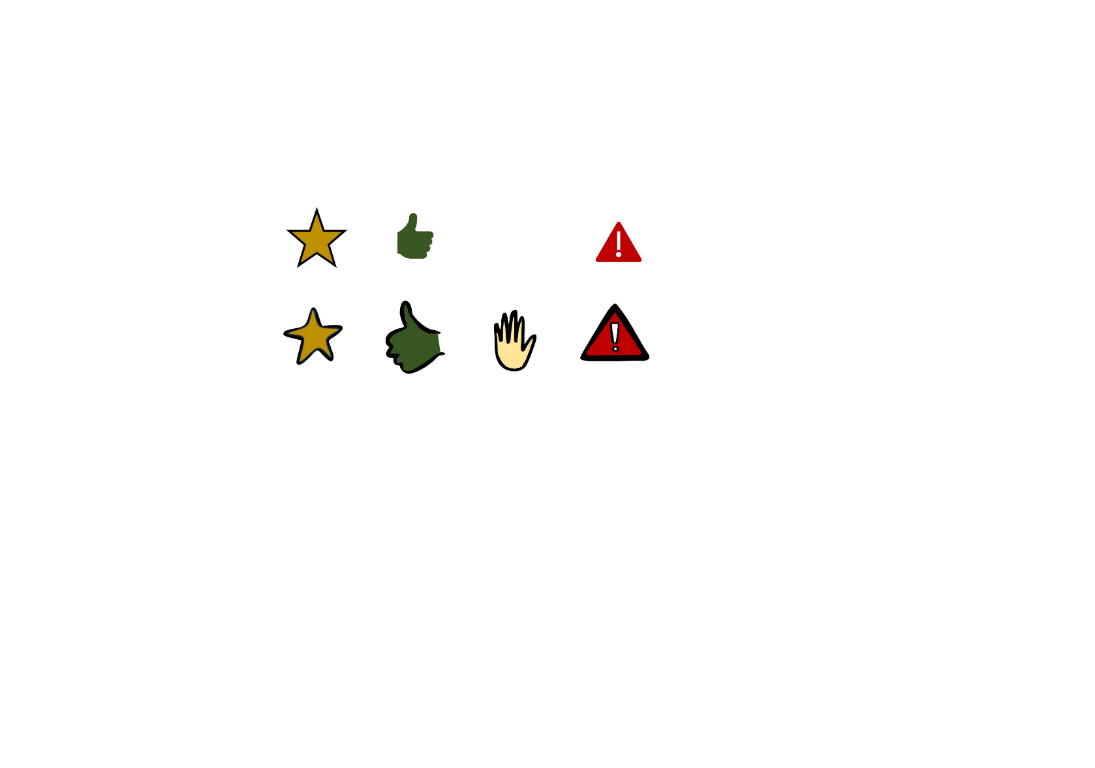 | 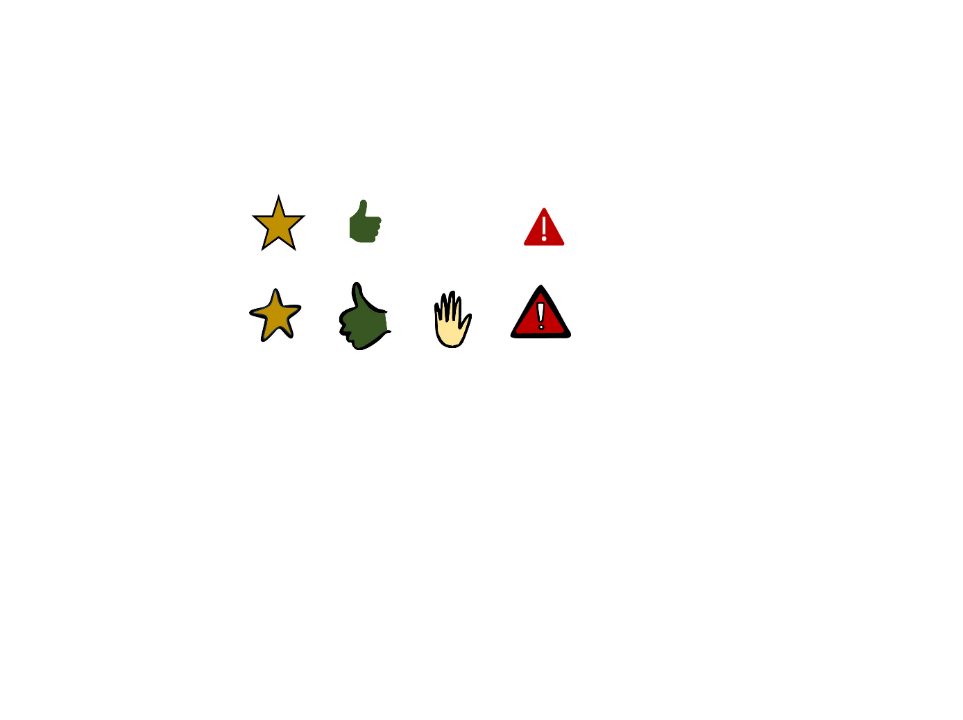 | 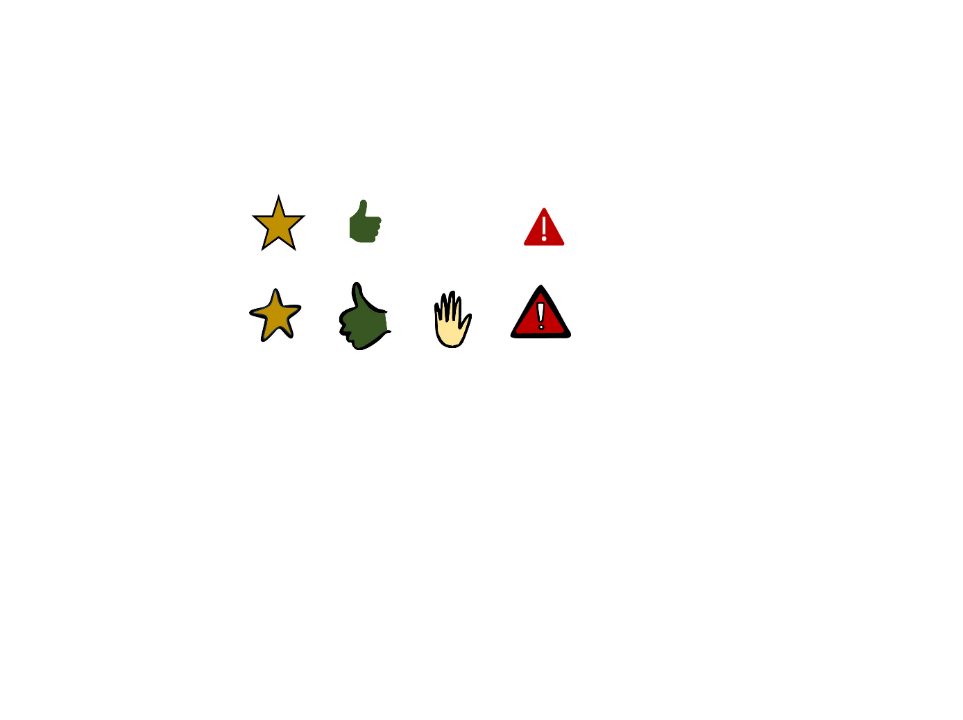 | |
|  |  |  |  |  |  |  |  |  |  |  |  |  |  |  |  |  |  |  | |
|  |  |  |  |  |  |  |  |  |  |  |  |  |  |  |  |  |  |  | |
| ^a^ Review Identifier; number: reference with meta-analysis (quantitative synthesis), letter: reference with narrative synthesis (qualitative synthesis) following O’Leary *et al.* 2017.  ^b^ Was the respective review already critically appraised by the CEESAT community and included in the CEEDER database?  **1.1** Are the elements of the review question clear?  **2.1** Is there an a-priori method/protocol document?  **3.1** Is the approach to searching clearly defined, systematic and transparent?  **3.2** Is the search comprehensive?  **4.1** Are eligibility criteria clearly defined?  **4.2** Are eligibility criteria consistently applied to all potentially relevant articles and studies found during the search?  **4.3** Are eligibility decisions transparently reported?  **5.1** Does the review critically appraise each study?  **5.2** During critical appraisal was an effort made to minimise subjectivity?  **6.1** Is the method of data extraction fully documented?  **6.2** Are the extracted data reported for each study?  **6.3** Were extracted data cross checked by more than one reviewer?  **7.1** Is the choice of synthesis approach appropriate?  **7.2** Is a statistical estimate of pooled effect (or similar) provided together with measure of variance and heterogeneity among studies?  **7.3** Is variability in the study findings investigated and discussed?  **8.1** Have the authors considered limitations of the synthesis? | | | | | | | | | | | | | | | | | | |  |

**Table A4.2** CEESAT ratings of all studies included in the summary table of scope.
