# Supplementary material for: What secondary research evidence exists on the effects of forest management after disturbances: a systematic map protocol
Source: Environ Evid. 2024 Jun 2;13:16. doi: 10.1186/s13750-024-00340-7 (PMC11378863; doi:10.1186/s13750-024-00340-7)
Supplement: Supplementary file 5 — Supplementary material 5. Test of comprehensiveness of the search results. [file 13750_2024_340_MOESM5_ESM.docx]

Supplementary Material

**Mapping secondary research evidence on the effects of forest management after disturbances -protocol for an overview of reviews**

Moritz Baumeister, Markus Meyer

**Additional file 5: Test of comprehensiveness of search results**

**Method:** We screened the first 20 Google Scholar search results of simple keyword searches in all four search categories for relevant articles based on their title. We used the following keywords: (1) salvage logging, ecosystem services, review, meta-analysis (2) salvage logging, biodiversity, review, meta-analysis (3) disturbance, tree planting, ecosystem services, review, meta-analysis (4) disturbance, tree planting, biodiversity, review, meta-analysis. Afterwards, we checked if all identified articles were covered by our search string when searching the data base Scopus. Both searches (Google Scholar, Scopus) were conducted on 27 March 2024.

All articles that were included in both searches are highlighted in green. If an article considered relevant was not included in the Scopus search but could be included by modifying the search string, it is highlighted in purple. Articles that remained uncovered by the search after modification of search strings are highlighted in red.

**Results:** The articles identified by the Google Scholar search as potentially relevant based on their title are listed below. More than half of the articles identified by the Google Scholar search were included in the Scopus search of the respective category. After modifications of search terms all relevant articles could be included in the Scopus search. The search terms that were added due to this test of comprehensiveness were: “tree regenerat*”, recov*, harvest* (a second time in the disturbance bracket), avian, datasets, degrad*, restorat* (a second time in the disturbance bracket), replace*

1. **Salvage logging and Ecosystem services**

Leverkus, Alexandro B., Lena Gustafsson, David B. Lindenmayer, Jorge Castro, José María Rey Benayas, Thomas Ranius, and Simon Thorn. "Salvage logging effects on regulating ecosystem services and fuel loads." *Frontiers in Ecology and the Environment* 18, no. 7 (2020): 391-400.

Leverkus, Alexandro B., Lena Gustafsson, José María Rey Benayas, and Jorge Castro. "Does post-disturbance salvage logging affect the provision of ecosystem services? A systematic review protocol." *Environmental Evidence* 4 (2015): 1-7.

Leverkus, Alexandro B., Inés Polo, Claire Baudoux, Simon Thorn, Lena Gustafsson, and Rafael Rubio de Casas. "Resilience impacts of a secondary disturbance: Meta‐analysis of salvage logging effects on tree regeneration." *Journal of Ecology* 109, no. 9 (2021): 3224-3232.

- “tree regenerat*”

Leverkus, Alexandro B., Brian Buma, Joseph Wagenbrenner, Philip J. Burton, Emanuele Lingua, Raffaella Marzano, and Simon Thorn. "Tamm review: Does salvage logging mitigate subsequent forest disturbances?." *Forest Ecology and Management* 481 (2021): 118721.

Taeroe, Anders, Johannes HC de Koning, Magnus Löf, Anne Tolvanen, Lárus Heiðarsson, and Karsten Raulund-Rasmussen. "Recovery of temperate and boreal forests after windthrow and the impacts of salvage logging. A quantitative review." *Forest Ecology and Management* 446 (2019): 304-316.

- recov*

1. **Salvage logging and Biodiversity**

Thorn, Simon, Claus Bässler, Roland Brandl, Philip J. Burton, Rebecca Cahall, John L. Campbell, Jorge Castro et al. "Impacts of salvage logging on biodiversity: A meta‐analysis." *Journal of Applied Ecology* 55, no. 1 (2018): 279-289.

Basile, Marco, Anton Krištín, Grzegorz Mikusiński, Simon Thorn, Michał Żmihorski, Gilberto Pasinelli, and Eckehard G. Brockerhoff. "Salvage logging strongly affects woodpecker abundance and reproduction: a meta-analysis." *Current Forestry Reports* 9, no. 1 (2023): 1-14.

Riffell, Sam, Jake Verschuyl, Darren Miller, and T. Bently Wigley. "Biofuel harvests, coarse woody debris, and biodiversity–a meta-analysis." *Forest Ecology and Management* 261, no. 4 (2011): 878-887.

- The term **harvest*** was added a second time in the disturbance bracket.

Bognounou, Fidle, L. A. Venier, S. L. Van Wilgenburg, Isabelle Aubin, J-N. Candau, A. Arsenault, Christian Hebert, Jacques Ibarzabal, S. J. Song, and Louis De Grandpré. "Early avian functional assemblages after fire, clearcutting, and post-fire salvage logging in North American forests." *Canadian Journal of Forest Research* 51, no. 3 (2021): 393-407.

- Outcome: **avian ;** study type: **datasets**

1. **Tree planting and Ecosystem services**

Shimamoto, Carolina Y., André A. Padial, Carolina M. da Rosa, and Márcia CM Marques. "Restoration of ecosystem services in tropical forests: A global meta-analysis." *PloS one* 13, no. 12 (2018): e0208523.

Benayas, José M. Rey, Adrian C. Newton, Anita Diaz, and James M. Bullock. "Enhancement of biodiversity and ecosystem services by ecological restoration: a meta-analysis." *science* 325, no. 5944 (2009): 1121-1124.

- Disturbance: degrad*

Ren, Yanjiao, Yihe Lü, Bojie Fu, and Kun Zhang. "Biodiversity and ecosystem functional enhancement by forest restoration: A meta‐analysis in China." *Land Degradation & Development* 28, no. 7 (2017): 2062-2073.

Hua, Fangyuan, L. Adrian Bruijnzeel, Paula Meli, Philip A. Martin, Jun Zhang, Shinichi Nakagawa, Xinran Miao et al. "The biodiversity and ecosystem service contributions and trade-offs of forest restoration approaches." *Science* 376, no. 6595 (2022): 839-844.

- The term **restorat*** was added a second time in the disturbance bracket

1. **Tree planting and Biodiversity**

Wang, Chao, Weiwei Zhang, Xiaona Li, and Juying Wu. "A global meta‐analysis of the impacts of tree plantations on biodiversity." *Global Ecology and Biogeography* 31, no. 3 (2022): 576-587.

Latawiec, Agnieszka E., Renato Crouzeilles, Pedro HS Brancalion, Ricardo R. Rodrigues, Jerˆonimo B. Sansevero, Juliana Silveira dos Santos, Morena Mills, Andre Gustavo Nave, and Bernardo B. Strassburg. "Natural regeneration and biodiversity: a global meta‐analysis and implications for spatial planning." *Biotropica* 48, no. 6 (2016): 844-855.

Kawamura, Kazuhiro, Yuichi Yamaura, Masashi Soga, Rebecca Spake, and Futoshi Nakamura. "Effects of planted tree species on biodiversity of conifer plantations in Japan: a systematic review and meta-analysis." *Journal of Forest Research* 26, no. 3 (2021): 237-246.

- Disturbance: **replace***
