# Supplementary material for: What secondary research evidence exists on the effects of forest management after disturbances: a systematic map protocol
Source: Environ Evid. 2024 Jun 2;13:16. doi: 10.1186/s13750-024-00340-7 (PMC11378863; doi:10.1186/s13750-024-00340-7)
Supplement: Supplementary file 6 — Supplementary material 6. Cohens Kappa Reviewer Agreement. [file 13750_2024_340_MOESM6_ESM.pdf]

Kappa_Values_Agreement_Reviewers

Moritz Baumeister

2024-03-30

Supplementary Material

What secondary research evidence exists on the effects of forest management after disturbances: a Systematic Map Protocol

Moritz Baumeister, Markus Meyer

Additional file 6: Assessment of Agreement between Reviewers

This script calculates example kappa values following Cohen 1960 and provides a Table to describe certain levels of agreement in words following Landis and Koch 1977

for additional examples see for instance the first example in Wikipedia Cohen's Kappa (accessed 30.03.2024) https://en.wikipedia.org/wiki/Cohen%27s_kappa

Cohen, J. (1960). A coefficient of agreement for nominal scales. Educational and psychological measurement, 20(1), 37-46.

Landis, J. R., & Koch, G. G. (1977). The measurement of observer agreement for categorical data. biometrics, 159-174.

```
#function with frequencies (following Cohen 1960)
k <- function(N, f_observed, f_chance) {(f_observed - f_chance) / (N - f_chance)}

#N: Number of decisions
#f_observed: How often did the reviewers agree?
#f_chance: What can be expected by chance with regard to agreement?
```

Kappa Statistic	Strength Agreement (after Landis and Koch 1977)	Example Agreement 100 Articles Screened
<0.00	Poor	40
0.00-0.20	Slight	55
0.21-0.40	Fair	65
0.41-0.60	Moderate	75
0.61-0.80	Substantial	85
0.81-1.00	Almost Perfect	95

```
#six examples

#The kappa values are narratively graded based on the "benchmarks"
```

```r
#in Landis and Koch 1977 (p.165)

#f_chance is 50 in all examples because 50 articles can be expected
#by chance alone to be excluded or included by both screeners simultaneously

#100 Papers are screened and the screeners agree in 95 cases regarding
#exclusion or inclusion (both "yes" or both "no")

k(100,95,50)# kappa = 0.9 "Almost perfect"
```

```
## [1] 0.9
```

```r
#100 Papers are screened and the screeners agree in 85 cases
#regarding exclusion or inclusion (both "yes" or both "no")

k(100,85,50)# kappa = 0.7 "Substantial"
```

```
## [1] 0.7
```

```r
#100 Papers are screened and the screeners agree in 75 cases
#regarding exclusion or inclusion (both "yes" or both "no")

k(100, 75,50)# kappa = 0.5 "Moderate"
```

```
## [1] 0.5
```

```r
#100 Papers are screened and the screeners agree in 65 cases
#regarding exclusion or inclusion (both "yes" or both "no")

k(100,65,50) # kappa = 0.3 "Fair"
```

```
## [1] 0.3
```

```r
#100 Papers are screened and the screeners agree in 55 cases
#regarding exclusion or inclusion (both "yes" or both "no")

k(100,55,50) # kappa = 0.1 "Slight"
```

```
## [1] 0.1
```

```r
#100 Papers are screened and the screeners agree in 40 cases
#regarding exclusion or inclusion (both "yes" or both "no")

k(100, 40, 50) # kappa = -0.2 "Poor"
```

```
## [1] -0.2
```
