# Supplementary material for: What secondary research evidence exists on the effects of forest management after disturbances: a systematic map protocol
Source: Environ Evid. 2024 Jun 2;13:16. doi: 10.1186/s13750-024-00340-7 (PMC11378863; doi:10.1186/s13750-024-00340-7)
Supplement: Supplementary file 7 — Supplementary material 7. Search Strings Web of Science and CABI Abstracts Forest Science Library. [file 13750_2024_340_MOESM7_ESM.docx]

|  |  |
| --- | --- |
| Supplementary Material  **What secondary research evidence exists on the effects of forest management after disturbances: a Systematic Map Protocol**  Moritz Baumeister, Markus Meyer  **Additional file 7: Search strings modified for Web of Science Core Collection and CABI Digital Library (Forest Science Collection)** | |
| **Category** | **Search String** |
| Salvage logging and ecosystem services | **Web of Science**  TS=(("forest*" OR "woodl*") AND ("disturb*" OR "succession*" OR "harvest*" OR "fire" OR "wildfire" OR "windthrow" OR "storm" OR (("pest" OR "insect*" OR "beetle*") AND ("outbreak" OR "attack"))) AND ("snag remov*" OR (("salvag*" OR "post*") AND ("log*" OR "harvest*" OR "cut*" OR "fell*"))) AND ("ecosystem service*" OR "environmental service*" OR "ecosystem function*" OR "wood supply" OR "tree regenerat*" OR "recov*" OR "water supply" OR "erosion" OR "pollinat*" OR "soil formation" OR "climate regulation" OR "carbon sequestration" OR "carbon storage" OR "recreat*" OR "microclimat*") AND (review OR "meta$analy*" OR "synthes*" OR "datasets"))  With additional specification of publication date:  2003-12-31 to 2024-12-31  **CABI Abstracts Forest Library**  ***within Abstracts***  (forest* OR woodl*) AND (disturb* OR succession* OR harvest* OR fire OR wildfire OR windthrow OR storm OR ((pest OR insect* OR beetle*) AND (outbreak OR attack))) AND  (“snag remov*” OR ((salvag* OR post*) AND (log* OR harvest* OR cut* OR fell*))) AND  (“ecosystem service*” OR “environmental service*” OR “ecosystem function*” OR “wood supply” OR “tree regenerat*” OR recov* OR “water supply” OR erosion OR pollinat* OR “soil formation” OR “climate regulation” OR “carbon sequestration” OR “carbon storage” OR recreat* OR microclimat*) AND  (review OR “meta$analy*” OR synthes* OR datasets)  With additional specification of publication date:  2003-12-31 to 2024-12-31 |
| Salvage logging and biodiversity | **Web of Science**  TS=((forest* OR woodl*) AND (disturb* OR succession* OR harvest* OR fire OR wildfire OR windthrow OR storm OR ((pest OR insect* OR beetle*) AND (outbreak OR attack))) AND (“snag remov*” OR ((salvag* OR post*) AND (log* OR harvest* OR cut* OR fell*))) AND (biodivers* OR diversity OR richness OR “species richness” OR “tree size diversity” OR microhabitat* OR “dead wood” OR invertebrat* OR vertebrat* OR flora OR vegetat* OR mammal* OR bird* OR avian OR reptile* OR amphibian*) AND (review OR “meta$analy*” OR synthes* OR datasets) )  With additional specification of publication date:  2003-12-31 to 2024-12-31  **CABI Abstracts Forest Library**  ***within Abstracts***  (forest* OR woodl*) AND (disturb* OR succession* OR harvest* OR fire OR wildfire OR windthrow OR storm OR ((pest OR insect* OR beetle*) AND (outbreak OR attack))) AND  (“snag remov*” OR ((salvag* OR post*) AND (log* OR harvest* OR cut* OR fell*))) AND  (biodivers* OR diversity OR richness OR “species richness” OR “tree size diversity” OR microhabitat* OR “dead wood” OR invertebrat* OR vertebrat* OR flora OR vegetat* OR mammal* OR bird* OR avian OR reptile* OR amphibian*) AND  (review OR “meta$analy*” OR synthes* OR datasets)  With additional specification of publication date:  2003-12-31 to 2024-12-31 |
| Tree planting (comparator: Natural regeneration) and ecosystem services | **Web of Science**  TS=( (forest* OR woodl*) AND (disturb* OR succession* OR degrad* OR restorat* OR log* OR harvest* OR cut* OR fell* OR fire OR wildfire OR windthrow OR storm OR ((pest OR insect* OR beetle*) AND (outbreak OR attack))) AND (plant* OR seed* OR sowing OR “seed* establish*” OR grow* OR afforestat* OR restorat* OR “natural rejuvenation” OR regenerat* OR rejuvenat* OR “seed* establish*” OR seed* OR “young adj4 tree*”) AND (“ecosystem service*” OR “environmental service*” OR “ecosystem function*” OR “wood supply” OR “tree regenerat*” OR recov* OR “water supply” OR erosion OR pollinat* OR “soil formation” OR “climate regulation” OR “carbon sequestration” OR “carbon storage” OR recreat* OR microclimat*) AND (review OR “meta$analy*” OR synthes*) )  With additional specification of publication date:  2003-12-31 to 2024-12-31  **CABI Abstracts Forest Library**  ***within Abstracts***  (forest* OR woodl*) AND (disturb* OR succession* OR degrad* OR restorat* OR log* OR harvest* OR cut* OR fell* OR fire OR wildfire OR windthrow OR storm OR ((pest OR insect* OR beetle*) AND (outbreak OR attack))) AND (plant* OR seed* OR sowing OR “seed* establish*” OR grow* OR afforestat* OR restorat* OR “natural rejuvenation” OR regenerat* OR rejuvenat* OR “seed* establish*” OR seed* OR “young adj4 tree*”) AND (“ecosystem service*” OR “environmental service*” OR “ecosystem function*” OR “wood supply” OR “tree regenerat*” OR recov* OR “water supply” OR erosion OR pollinat* OR “soil formation” OR “climate regulation” OR “carbon sequestration” OR “carbon storage” OR recreat* OR microclimat*) AND (review OR “meta$analy*” OR synthes*)  With additional specification of publication date:  2003-12-31 to 2024-12-31 |
| Tree planting (comparator: Natural regeneration) and biodiversity | **Web of Science**  TS=((forest* OR woodl*) AND (disturb* OR succession* OR degrad* OR restorat* OR replace* OR log* OR harvest* OR cut* OR fell* OR fire OR wildfire OR windthrow OR storm OR ((pest OR insect* OR beetle*) AND (outbreak OR attack))) AND  (plant* OR seed* OR sowing OR “seed* establish*” OR grow* OR afforestat* OR restorat* OR “natural rejuvenation” OR regenerat* OR rejuvenat* OR “seed* establish*” OR seed* OR “young adj4 tree*”) AND  (biodivers* OR diversity OR richness OR “species richness” OR “tree size diversity” OR microhabitat* OR “dead wood” OR invertebrat* OR vertebrat* OR flora OR vegetat* OR mammal* OR bird* OR avian OR reptile* OR amphibian*) AND  (review OR “meta$analy*” OR synthes*))  With additional specification of publication date:  2003-12-31 to 2024-12-31  **CABI Abstracts Forest Library**  ***within Abstracts***  (forest* OR woodl*) AND (disturb* OR succession* OR degrad* OR restorat* OR replace* OR log* OR harvest* OR cut* OR fell* OR fire OR wildfire OR windthrow OR storm OR ((pest OR insect* OR beetle*) AND (outbreak OR attack))) AND (plant* OR seed* OR sowing OR “seed* establish*” OR grow* OR afforestat* OR restorat* OR “natural rejuvenation” OR regenerat* OR rejuvenat* OR “seed* establish*” OR seed* OR “young adj4 tree*”) AND (biodivers* OR diversity OR richness OR “species richness” OR “tree size diversity” OR microhabitat* OR “dead wood” OR invertebrat* OR vertebrat* OR flora OR vegetat* OR mammal* OR bird* OR avian OR reptile* OR amphibian*) AND (review OR “meta$analy*” OR synthes*)  With additional specification of publication date:  2003-12-31 to 2024-12-31 |
